# Micellar effects upon synthesis of 1,2-dihydro-1-arylnaphtho[1,2-*e*][1,3]oxazine-3-ones in water at room temperature

**DOI:** 10.1039/d5ra08594b

**Published:** 2026-05-19

**Authors:** Saeedeh Asadian, Mohsen Moradian, Javad Safari

**Affiliations:** a Department of Organic Chemistry, Faculty of Chemistry, University of Kashan Kashan P.O. Box 87317-51167 Iran m.moradian@kashanu.ac.ir

## Abstract

In this study, the efficient synthesis of naphthoxazinones *via* a three-component reaction of β-naphthol, benzaldehyde, and urea in the presence of the micellar catalyst sodium dodecyl sulfate (SDS) in an aqueous medium was comprehensively investigated. The reactions were performed under both room temperature and ultrasonic conditions, yielding satisfactory results in both cases. However, the use of ultrasound resulted in an increased yield and a remarkable reduction in reaction time. The micellar structure of SDS creates a hydrophobic microenvironment that concentrates the nonpolar β-naphthol and benzaldehyde molecules in the micelle core. At the same time, the highly polar urea remains in the surrounding aqueous phase, with the reaction occurring at the interface between these two phases. This molecular arrangement increases the local concentration of reactants and lowers the activation energy, thereby facilitating the condensation process. In addition to its high yield, this method offers significant environmental advantages because it uses water as the solvent.

## Abstract 2

An efficient micelle-promoted synthesis of 12-dihydro-1-arylnaphtho-, an efficient micelle-promoted synthesis of 1,2-dihydro-1-arylnaphtho [1,2-*e*]-[1,3]oxazine-3-ones in water at room temperature with broad substrate scope is described.

## Graphical abstract

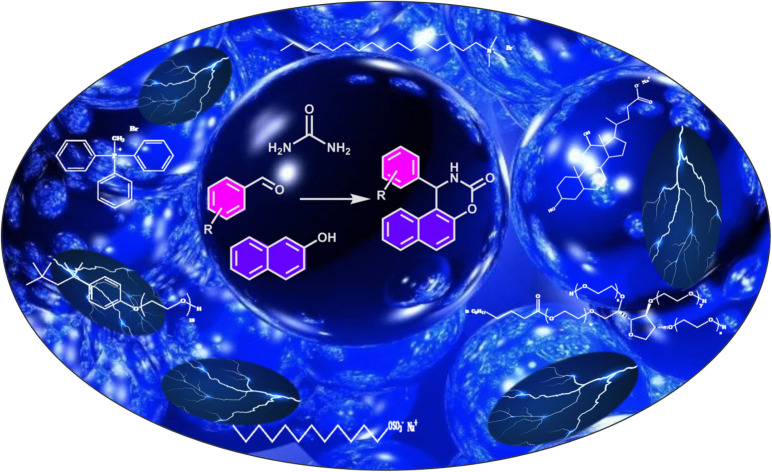

## 1 Introduction

Since the emergence of green chemistry, significant advances have been made in replacing petroleum-based chemicals with bio-based alternatives.^1,2^ This trend aims to reduce environmental impacts and enhance the sustainability of chemical processes, prioritizing the development of novel and efficient approaches that improve reaction efficacy while maintaining environmental compatibility. Among the twelve principles of green chemistry, the choice of appropriate solvents plays a key role in minimizing toxicity, energy consumption, and environmental pollution.^3–5^

Although water is recognized as a safe, accessible, and environmentally friendly solvent, limitations such as the poor solubility of many organic compounds and the instability of sensitive materials and catalysts in aqueous media still justify the widespread use of organic solvents. These challenges have motivated the development of methods that enable efficient organic reactions to be conducted in aqueous environments.^6,7^

**Scheme 1:**
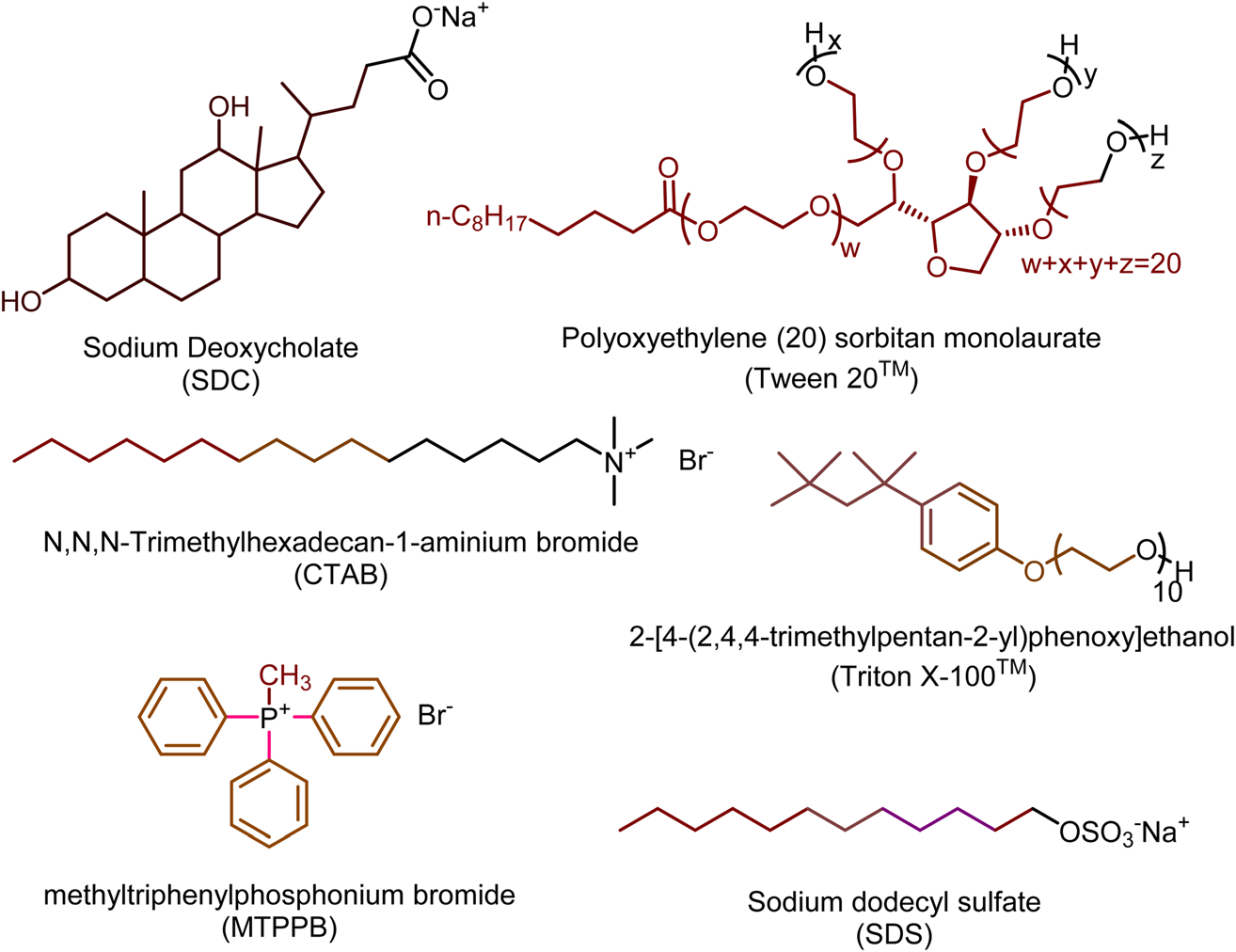
The structure of common surfactants.

Surfactants, as amphiphilic compounds with dual structures consisting of a polar hydrophilic head and a nonpolar hydrophobic tail, reduce surface tension between different phases and facilitate the balance between aqueous and non-aqueous media.^8–11^ Depending on the nature of their polar groups, these molecules are classified into anionic, cationic, nonionic, and amphoteric categories, each exhibiting distinct properties and applications.^12^ Examples of surfactants are presented in Scheme 1.

One of the prominent applications of surfactants is their role as catalysts through the phenomenon of micellar catalysis. In this process, amphiphilic molecules with various charges aggregate at low critical concentrations in aqueous media to form micellar structures. These structures orient the polar sections toward the aqueous environment, while concentrating the nonpolar sections in the micelle core, creating a hydrophobic microenvironment that hosts organic compounds and thereby increases solubility, facilitating organic reactions.^12–15^ Micellar catalysis significantly enhances reaction rates by lowering activation energy and increasing selectivity and is recognized as an effective and environmentally friendly approach in organic synthesis.^16^

Catalysts, which remain chemically unchanged and stable at the end of a reaction, play a fundamental role in the kinetic analysis of chemical reactions by facilitating reaction pathways and lowering activation energies.^17,18^ Their application has gained particular significance in accelerating multicomponent reactions (MCRs), which are recognized as efficient, rapid, and selective methods for synthesizing complex compounds.^19^

Multicomponent reactions involve the simultaneous reaction of multiple reactants in a single one-pot system to produce a unique product with a complex structure.^20^ Their notable advantages include reduced reaction times, minimized by-products, elimination of multiple purification steps, cost reduction, utilization of readily available starting materials, and operational simplicity—features that align well with the principles of green chemistry.^21,22^ In recent years, MCRs have emerged as powerful synthetic tools, especially in the construction of heterocyclic compounds with complex architectures, leading to innovative strategies that enhance synthesis efficiency and yield.^23^

Arylnaphthoxazine-3-ones are an important class of heterocyclic compounds due to many of their derivatives exhibiting biological activities. These include functioning as HIV-1 reverse transcriptase inhibitors^24^ and demonstrating *in vitro* anticancer activity against breast (MCF-7) and colon (HCT116) cancer cell lines.^25^ Additionally, the naphthoxazine core structure has been investigated for its antimicrobial^26^ and antifungal properties,^27^ making it a valuable template for developing new anti-infective agents. This compound is particularly useful as a building block for synthesizing a range of diverse heterocyclic systems, including pyrimidines, pyrazoles, and isoxazoles, which are recognized as privileged structures in pharmaceutical development.^28–30^

1,3-Oxazines are six-membered heterocycles comprising oxygen and nitrogen atoms at positions 1 and 3, respectively. In some derivatives, a carbonyl group is present on a ring carbon, forming ketone-containing structures such as oxazinones.^31,32^ Among the most notable derivatives are 1-aryl-1,2-dihydronaphtho[1,2-*e*][1,3]oxazin-3-ones, which possess a fused-ring system where a naphthalene moiety is attached to the oxazine scaffold. Due to their spatial stability and ability to engage in targeted interactions with proteins and enzymes, these compounds exhibit diverse biological activities, including antimicrobial, anticancer, and anti-inflammatory effects.^33,34^

The synthesis of 1-aryl-1,2-dihydronaphtho[1,2-*e*][1,3]oxazin-3-ones from β-naphthol, aldehydes, and urea is carried out *via* a cyclocondensation reaction within the framework of one-pot multicomponent reactions. In the synthesis of these compounds, various effective catalysts such as pyridinium-based ionic liquids,^35^ cellulose sulfuric acid,^36^ Amberlite IRA-400 Cl resin 37, perlite-SO_3_H nanoparticles,^38,39^, [PyPS][HSO_4_],^40^ TMSCl,^41^ Cu nanoparticles,^42^ Zn(OTf)_2_,^43^ HClO_4_/SiO_2_,^44^, [bmim]Br,^45^ TMSCl/NaI,^46^ ZnO nanoparticles^47^ and ompg-C_3_N_4_/SO_3_H^48^ have been employed. Although these methods demonstrate efficacy, some face challenges, including prolonged reaction times, the use of toxic solvents, high temperatures, and low yields.

**Scheme 2:**
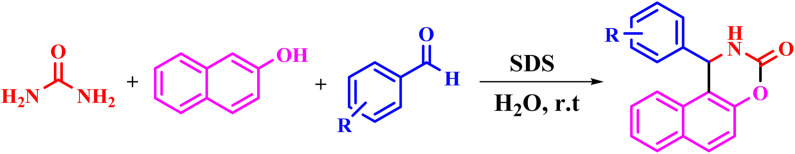
Synthesis of 1-aryl-1,2-dihydronaphtho[1,2-*e*][1,3]oxazin-3-ones (4a–l) in the presence of SDS as a catalyst.

In this study, a green chemistry approach has been introduced for synthesizing derivatives of 1-aryl-1,2-dihydronaphtho[1,2-*e*][1,3]oxazin-3-ones. The process employs sodium dodecyl sulfate (SDS) as a catalyst in an aqueous medium under ultrasonic irradiation at ambient temperature (Scheme 2). These reaction conditions accelerate product formation and achieve high yields within a short time, demonstrating the high efficiency of the proposed system alongside its eco-friendly nature and operational simplicity.

## 2 Experimental

### 2.1. Chemicals and instrumentation

The raw materials and solvents required were procured from reputable commercial suppliers. Thin-layer chromatography (TLC) using silica gel plates (Silica sheets FG_245_) was employed to monitor the progress of the reactions. Structural characterization of the compounds was performed by nuclear magnetic resonance (NMR) spectroscopy using a Bruker Avance DRX spectrometer operating at 400 MHz for ^1^H and 100 MHz for ^13^C nuclei, with spectra recorded in deuterated DMSO solvent. Chemical shifts (*δ*) were reported in parts per million (ppm) relative to tetramethylsilane (TMS) as an internal standard, and coupling constants (*J*) were given in hertz (Hz). Additionally, Fourier-transform infrared (FT-IR) spectroscopy (Magana 550, Nicolet) was utilized to characterize both the catalyst and the synthesized products. Melting points of the samples were determined using a Scientific Thermo MK3IA 9300 apparatus (England). Collectively, these analytical techniques enabled a comprehensive and precise evaluation of the physical and chemical properties of the synthesized materials.

### 2.2. General approach for the preparation of 1,2-dihydro-1-arylnaphtho[1,2-*e*][1,3]oxazine-3-ones derivatives using ultrasonic irradiation

A mixture of β-naphthol (1 mmol), benzaldehyde (1 mmol), urea (1 mmol) and sodium dodecyl sulfate (SDS) (10 mol%) was added to a 25 mL round-bottom flask containing 5 mL water, and the resulting suspension was subjected to ultrasonic irradiation at 80 W. The reaction progress was continuously monitored by thin-layer chromatography (TLC) employing a solvent system of ethyl acetate and *n*-hexane in a 3 : 7 ratio. Upon completion of the reaction within the specified time frame, the resulting solid product was isolated *via* filtration. To remove impurities, the solid was sequentially washed with hot water. The crude product was purified through recrystallization from ethanol, effectively obviating the need for further refinement steps.

### 2.3. General approach for the preparation of 1,2-dihydro-1-arylnaphtho[1,2-*e*][1,3]oxazine-3-ones derivatives by thermal condition

In a 25 mL round-bottom flask containing 5 mL of water, 1 mmol of β-naphthol, 1 mmol of benzaldehyde, 1 mmol of urea, and 15 mol% sodium dodecyl sulfate (SDS) were added and the mixture was stirred continuously at room temperature. The progress of the reaction was monitored by TLC, and upon completion, the solid product was separated by filtration and washed with hot water (3 × 10 ml), and cold ethanol (3 × 5 ml). Finally, for further purification, the product was recrystallized from hot ethanol.

### 2.4. Spectral data of products

#### 2.4.1 1-Phenyl-1,2-dihydro-naphtho[1,2-*e*][1,3]oxazin-3-one (4a)

Yield = 248 mg (90%); mp_obs_ = 210–222 °C; ^1^H NMR (400 MHz, DMSO-*d*_6_): 8.934 (d, *J* = 2.8 Hz, 1H, NH), 7.822 (d, *J* = 7.6 Hz, 1H, Ar–H), 7.762 (d, *J* = 8.8 Hz, 2H, Ar–H), 7.238–7.168 (m, 8H, Ar–H), 6.275 (d, *J* = 3.2 Hz, 1H, CH); IR (KBr) 3447, 3405, 3357, 3209, 1643, 1441, 1352, 1266, 1171, 815 cm^−1^.

#### 2.4.2 1-(4-Chlorophenyl)-1,2-dihydro-naphtho[1,2-*e*][1,3]oxazin-3-one (4b)

Yield = 300 mg (97%); mp_obs_ = 212–214 °C; ^1^H NMR (400 MHz, DMSO-*d*_6_) (*δ* ppm): 8.906 (d, *J* = 2.8 Hz, 1H, NH), 8.00 (d, *J* = 8.8 Hz, 1H, Ar–H), 7.959 (d, *J* = 8.8 Hz, 1H, Ar–H), 7.796 (d, *J* = 8.0 Hz, 1H, Ar–H), 7.519–7.439 (m, 2H, Ar–H), 7.416–7.377 (m, 3H, Ar–H), 7.334 (d, *J* = 8.8 Hz, 2H, Ar–H), 6.250 (d, *J* = 3.2 Hz, 1H, CH); IR (KBr) 3424, 3208, 3140, 2967, 1748, 1629, 1508, 1387, 1223, 1123, 1081, 927, 789 cm^−1^.

#### 2.4.3 1-(2-Nitrophenyl)-1,2-dihydro-naphtho[1,2-*e*][1,3]oxazin-3-one (4c)

Yield = 304 mg (95%); mp_obs_ = 104–106 °C; ^1^H NMR (400 MHz, DMSO-*d*_6_) (*δ* ppm): 9.044 (d, *J* = 2.4 Hz, 1H, NH), 8.334–8.264 (m, 1H, Ar–H), 8.140 (d, *J* = 8.0 Hz, 1H, Ar–H), 8.041 (d, *J* = 8.8 Hz, 1H, Ar–H), 7.971 (d, *J* = 8.0 Hz, 1H, Ar–H), 7.854 (d, *J* = 8.0 Hz, 1H, Ar–H), 7.681–7.600 (m, 2H, Ar–H), 7.528–7.418 (m, 3H, Ar–H), 6.487 (d, *J* = 2.8 Hz, 1H, CH); IR (KBr) 3257, 3159, 2927, 1752, 1632, 1526, 1345, 1222, 1175, 1099, 920, 818, 745 cm^−1^.

#### 2.4.4 1-(3-Chlorophenyl)-1,2-dihydro-naphtho[1,2-*e*][1,3]oxazin-3-one (4d)

Yield = 297 mg (96%); mp_obs_ = 228–231 °C; ^1^H NMR (400 MHz, DMSO-*d*_6_) (*δ* ppm): 8.935 (d, *J* = 2.8 Hz, 1H, NH), 8.018 (d, *J* = 9.2 Hz, 1H, Ar–H), 7.967 (d, *J* = 8.0 Hz, 1H, Ar–H), 7.829 (d, *J* = 8.0 Hz, 1H, Ar–H), 7.537–7.447 (m, 3H, Ar–H), 7.399 (d, *J* = 9.2 Hz, 1H, Ar–H), 7.373–7.328 (m, 2H, Ar–H), 7.205–7.162 (m, 1H, Ar–H), 6.275 (d, *J* = 3.2 Hz, 1H, CH); IR (KBr): 3244, 3118, 2974, 1711, 1648, 1479, 1224, 1175, 1092, 784 cm^−1^.

#### 2.4.5 1-(3-Nitrophenyl)-1,2-dihydronaphtho[1,2-*e*][1,3]oxazin-3-one (4e)

Yield = 300 mg (94%); mp_obs_ = 210–212 °C; ^1^H NMR (400 MHz, DMSO-*d*_6_) (*δ* ppm): 9.048 (d, *J* = 2.8 Hz, 1H, NH), 8.340–8.266 (m, 1H, Ar–H), 8.159–8.129 (m, 1H, Ar–H), 8.044 (d, *J* = 7.6 Hz, 1H, Ar–H), 7.853 (d, *J* = 7.6 Hz, 1H, Ar–H), 7.682–7.602 (m, 2H, Ar–H), 7.531–7.419 (m, 3H, Ar–H), 6.487 (d, *J* = 3.2 Hz, 1H, CH); IR (KBr) 3421, 3316, 3059,2922,1622, 1511, 1393, 1277, 1111, 1035, 936 cm^−1^.

#### 2.4.6 1-(4-Iso-propylphenyl)-1,2-dihydro-naphtho[1,2-*e*][1,3]oxazin-3-one (4f)

Yield = 275 mg (87%); mp_obs_ = 240–242 °C; ^1^H NMR (400 MHz, DMSO-*d*_6_): 8.819 (d, *J* = 2.4 Hz, 1H, NH), 8.128–7.939 (m, 2H, Ar–H), 7.837 (d, *J* = 8.0 Hz, 1H, Ar–H), 7.513–7.428 (m, 2H, Ar–H), 7.380 (d, *J* = 8.8 Hz, 1H, Ar–H), 7.336–7.141 (m, 4H, Ar–H), 6.150 (d, *J* = 2.8 Hz, 1H, CH), 2.855–2.754 (septet, 1H, CH), 1.132 (d, *J* = 2.4 Hz, 3H, CH_3_), 1.115 (d, *J* = 2.4 Hz, 3H, CH_3_); IR (KBr): 3424, 3208, 3140, 2967, 1748, 1629, 1508, 1387, 1223, 1123, 1081, 927, 789 cm^−1^.

#### 2.4.7 1-(4-Methoxyphenyl)-1,2-dihydro-naphtho [1,2-*e*][1,3] oxazin-3-one (4g)

Yield = 262 mg (86%); mp_obs_ = 188–190 °C; ^1^H NMR (400 MHz, DMSO-*d*_6_): 8.793 (d, *J* = 2.4 Hz, 1H, NH), 7.985 (d, *J* = 9.2 Hz, 1H, Ar–H), 7.836 (d, *J* = 8.0 Hz, 1H, Ar–H), 7.536–7.444 (m, 4H, Ar–H), 6.763 (d, *J* = 8.8 Hz, 2H, Ar–H), 6.303 (d, *J* = 8.8 Hz, 2H, Ar–H), 6.270 (d, *J* = 2.4 Hz, 1H, CH), 3.810 (s, 3H, OCH_3_); IR (KBr) 3224, 3147, 2953, 2839, 1735, 1606, 1510, 1388, 1250, 1174, 1027, 923, 825, 747 cm^−1^.

#### 2.4.8 1-(2,4-Dichlorophenyl)-1,2-dihydronaphtho[1,2-*e*][1,3]oxazin-3-one (4h)

Yield = 316 mg (92%); mp_obs_ = 215–216 °C; ^1^H NMR (400 MHz, DMSO-*d*_6_): 8.910 (d, *J* = 1.6 Hz, 1H, NH), 8.028 (d, *J* = 9.2 Hz, 1H, Ar–H), 7.968 (d, *J* = 8.0 Hz, 1H, Ar–H), 7.704 (d, *J* = 2.0 Hz, 1H, Ar–H), 7.536–7.451 (m, 3H, Ar–H), 7.397–7.360 (m, 2H, Ar–H), 7.233 (d, *J* = 8.4 Hz, 1H, Ar–H), 6.517 (d, *J* = 2.4 Hz, 1H, CH); IR (KBr) 3429, 3244, 3141, 1736, 1629, 1514, 1389, 1226, 1180, 1027, 988, 830, 744 cm^−1^.

#### 2.4.9 1-(2-Chlorophenyl)-1,2-dihydronaphtho [1,2-*e*][1,3] oxazin-3-one (4i)

Yield = 290 mg (94%); mp_obs_ = 248–250 °C; ^1^H NMR (400 MHz, DMSO-*d*_6_): 8.750 (d, *J* = 2.8 Hz, 1H, NH), 7.885 (d, *J* = 8.8 Hz, 1H, Ar–H), 7.601 (d, *J* = 8.0 Hz, 1H, Ar–H), 7.389–7.301 (m, 3H, Ar–H), 7.240–7.140 (m, 3H, Ar–H), 6.951 (d, *J* = 8.8 Hz, 1H, Ar–H), 6.282 (d, *J* = 3.2 Hz, 1H, CH); IR (KBr) 3416, 3235, 3135, 2950, 1722, 1393, 1225, 1120, 823, 748 cm^−1^.

#### 2.4.10 1-(4-Dimethylaminophenyl)-1,2-dihydronaphtho[1,2-*e*][1,3]oxazin-3-one (4j)

Yield = 274 mg (86%); mp_obs_ = 223–224 °C; ^1^H NMR (400 MHz, DMSO-*d*_6_): 8.702 (d, *J* = 2.8 Hz, 1H, NH), 7.973–7.927 (m, 2H, Ar–H), 7.797 (d, *J* = 8.0 Hz, 1H, Ar–H), 7.493–7.419 (m, 2H, Ar–H), 7.359 (d, *J* = 9.2 Hz, 1H, Ar–H), 7.093 (d, *J* = 8.8 Hz, 2H, Ar–H), 6.633 (d, *J* = 8.8 Hz, 2H, Ar–H), 6.042 (d, *J* = 2.8 Hz, 1H, CH), 2.820 (s, 6H, CH_3_); IR (KBr) 3220, 3150, 2952, 2800, 1734, 1609, 1518, 1387, 1219, 1173, 916, 817, 740 cm^−1^.

#### 2.4.11 1-(3,4-Dimethoxyphenyl)-1,2-dihydro-naphtho[1,2-*e*][1,3]oxazin-3-one (4k)

Yield = 285 mg (85%); mp_obs_ = 189–190 °C; ^1^H NMR (400 MHz, DMSO-*d*_6_): 8.786 (d, *J* = 2.4 Hz, 1H, NH), 7.987 (d, *J* = 8.8 Hz, 1H, Ar–H), 7.949 (d, *J* = 7.6 Hz, 1H, Ar–H), 7.821 (d, *J* = 8.0 Hz, 1H, Ar–H), 7.510–7.431 (m, 2H, Ar–H), 7.377 (d, *J* = 8.8 Hz, 1H, Ar–H), 7.07 (d, *J* = 1.6 Hz, 1H, Ar–H), 6.838 (d, *J* = 8.4 Hz, 1H, Ar–H), 6.615 (dd, *J*_1_ = 8.4 Hz, *J*_2_ = 2.0 Hz, 1H, Ar–H), 6.135 (d, *J* = 3.2 Hz, 1H, CH), 3.722 (s, 3H, CH_3_), 3.672 (s, 3H, CH_3_); IR (KBr) 3429, 3226, 3148, 2948, 1734, 1515, 1393, 1227, 1135, 1025, 926, 812, 742 cm^−1^.

#### 2.4.12 1-(3-Methoxyphenyl)-1,2-dihydro-naphtho [1,2-*e*][1,3] oxazin-3-one (4l)

Yield = 268 mg (88%); mp_obs_ = 186–188 °C; ^1^H NMR (400 MHz, DMSO-*d*_6_): 8.859 (d, *J* = 1.6 Hz, 1H, NH), 7.996 (d, *J* = 9.2 Hz, 1H, Ar–H), 7.840 (d, *J* = 8.0 Hz, 1H, Ar–H), 7.520–7.437 (m, 2H, Ar–H), 6.958 (s, 1H, Ar–H), 6.843 (d, *J* = 8.0 Hz, 1H, Ar–H), 6.786 (d, *J* = 7.6 Hz, 1H, Ar–H), 6.172 (d, *J* = 2.4 Hz, 1H, CH), 3.712 (s, 3H, OCH_3_); IR (KBr) 3210, 3132, 2961, 1746, 1606, 1596, 1391, 1224, 1178, 1037, 928, 803, 744 cm^−1^.

## 3 Results and discussion

In an effort to develop a novel, efficient, and environmentally friendly method for the synthesis of naphthoxazinone derivatives, the three-component reaction of β-naphthol, benzaldehyde, and urea was investigated in an aqueous medium and in the presence of various surfactants under both thermal and ultrasonic conditions.

The critical micelle concentration (CMC) is the minimum concentration of a surfactant in water at which surfactant molecules form micelles. Below the CMC, surfactants are dispersed as individual molecules. Above this concentration, they self-assemble into spherical micelles. The alkyl chain length, type of polar group, and environmental conditions, including pH, temperature, and the presence of electrolytes, influence the CMC value in surfactants. In this research, it is crucial to pay attention to the CMC values of the surfactants used. The catalytic effect of the surfactant appears when it forms micelles, which means that the concentration of the surfactant in the reaction medium must be lower than its CMC value.

To achieve optimal conditions, the effects of several factors including the type and amount of surfactant, reaction temperature, and ultrasound power on the reaction yield and rate were carefully evaluated. Based on the results obtained from the optimization, the synthesis of derivatives was carried out under the selected conditions.

### 3.1. Optimization of the reaction conditions

To determine the optimal conditions for the efficient synthesis of naphthoxazinone derivatives, the three-component reaction of β-naphthol, urea, and benzaldehyde was systematically investigated in aqueous medium and in the presence of various surfactants under both thermal and ultrasonic conditions. All thermal reactions were monitored under identical conditions, with yields recorded after 22 minutes to allow for a meaningful comparison of surfactant efficiency, regardless of reaction completion. This method evaluates the relative catalytic activity of different surfactants within the same timeframe. The results of these studies are presented in detail in Table 1. In the first stage, the effect of surfactant type on the reaction yield under thermal conditions (room temperature and 15 mol% surfactant) was evaluated. According to the data in Table 1, the use of sodium dodecyl sulfate (SDS) as the surfactant afforded the highest yield (93%) within 23 minutes (Table 1, Entry 7). Other surfactants, such as SDC, MTPDB, CTAB, Tween 20, and Triton X-100, gave yields of 65%, 52%, 42%, 38%, and 25%, respectively (Table 1, Entries 2–6). Notably, in the absence of any surfactant, the reaction showed no significant progress (Table 1, Entry 1). These findings clearly highlight the crucial role of the surfactant's structure and nature in facilitating the reaction and enhancing the yield. Subsequently, the effects of different amounts of SDS (10, 15, and 20 mol%) as well as reaction temperature were examined. The results showed that decreasing the amount of SDS to below 15 mol% or increasing it above this value both led to a reduction in reaction yield (Table 1, Entries 8 and 9).

**Table 1:** Investigation of the effects of various parameters on the synthesis of 1,2-dihydro-1-arylnaphtho [1,2-*e*]-[1,3]oxazine-3-ones at some surfactants in water^d^

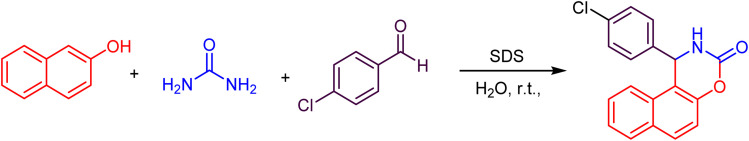									
Entry	Surfactant	Thermal condition^a^				US condition^b^			
		Surfactant (mol%)	Temp. (°C)	Time (min)	Yield^c^ (%)	Surfactant (mol%)	Power (W)	Time (min)	Yield^c^ (%)
1	—	—	25	22	—	—	80	10	—
2	Triton X-100	15	25	22	25	10	80	10	32
3	Tween 20	15	25	22	38	10	80	10	44
4	cTAB	15	25	22	42	10	80	10	50
5	MTPDB	15	25	22	52	10	80	10	58
6	SDC	10	25	22	65	10	80	10	72
7	SDS	**15**	**25**	**22**	**93**	**10**	**80**	**10**	**97**
8	SDS	20	25	22	93	5	80	10	53
9	SDS	10	50	22	43	15	80	10	97
10	SDS	15	50	22	53	10	40	10	45
11	SDS	15	80	22	37	10	60	10	77
12	SDS	15	100	22	Trace	10	100	10	83

a Thermal condition.

b Ultrasonic irradiation.

c Isolate yields.

d Reaction conditions: β-naphthol (1 mmol) with 4-chlorobenzaldehyde (1 mmol) and urea (1 mmol) in water.

Moreover, it was observed that increasing the reaction temperature (50, 80 and 100 °C) led to a significant decrease in product yield (Table 1, Entries 10–12). This phenomenon can be explained by the temperature-dependent micellization behavior of the surfactant, as investigated by Kumar *et al.*.^44^ According to their results, the critical micelle concentration (CMC) exhibits a temperature dependence. At higher temperatures, the CMC increases, indicating that a higher concentration of surfactant is required to form micelles, which effectively reduces the number of active micellar domains in the media. Moreover, the thermodynamic analysis reveals a shift in the micellization process from being entropy-driven at lower temperatures to enthalpy-driven at higher temperatures, reflecting a reduction in disorder-driven aggregation and greater thermal sensitivity.^44^ These findings explain the observed decrease in catalytic performance at elevated temperatures.

In the second part of the study, the effect of ultrasound irradiation on the three-component reaction was investigated. For this purpose, the reaction was performed with 10 mol% of each surfactant under ultrasound irradiation at 80 W. Specifically, the use of SDS as the surfactant under these conditions afforded the highest yield (97%) in only 10 minutes, representing a significant improvement over the thermal conditions (Table 1, Entry 7). Other surfactants also showed increased yields and reduced reaction times compared to the thermal method (Table 1, Entries 2–6). These results clearly demonstrate the pronounced effect of ultrasound in accelerating the reaction and improving the yield, particularly when SDS is used as the optimal surfactant. Furthermore, evaluation of different SDS amounts (5, 10, and 15 mol%) under ultrasound conditions revealed that 10 mol% SDS provided the best performance and higher yield compared to other amounts (Table 1, Entries 7–9). In addition, the three-component reaction was examined under ultrasound at various powers (40, 60, 80, and 100 W), and the results indicated that increasing the ultrasound power up to 80 W led to the highest yield and shortest reaction time (Table 1, Entries 7 and 10–12).

The results summarized in Table 1 clearly demonstrate that the use of sodium dodecyl sulfate (SDS) as a micellar surfactant in an aqueous medium provides optimal conditions for the synthesis of naphthoxazinone derivatives. According to the obtained data, the optimal reaction conditions under thermal conditions involve using 15 mol% SDS at 25 °C (Table 1, Entry 7), which yields a significant efficiency. In contrast, under ultrasonic conditions, the best performance was achieved using 10 mol% SDS combined with ultrasound irradiation at 80 W (Table 1, Entry 7). To enable a direct and fair comparison between the thermal and ultrasonic methods, the SDS concentration was set to 10 mol% in both cases to ensure identical conditions for performance evaluation. These conditions were subsequently employed as the basis for the synthesis of other derivatives in this study.

Based on the data presented in Table 2, the investigation of various substituents on the benzaldehyde ring indicates that electron-withdrawing groups, by increasing the reactivity of the carbonyl group, lead to higher yields and shorter reaction times in the synthesis of naphthoxazinones. In contrast, electron-donating groups decrease the reactivity of benzaldehyde, resulting in lower yields and longer reaction times. Halogen substituents also exhibit behavior similar to electron-withdrawing groups, promoting the reaction with high yields and short reaction times. Moreover, benzaldehyde demonstrates intermediate performance in terms of yield and reaction time. Moreover, in all cases, the use of ultrasonic conditions compared to room temperature conditions has significantly improved the yield and reduced the reaction time. These findings clearly highlight the decisive role of substituent type and reaction conditions in enhancing the efficiency of micellar synthesis of naphthoxazinones.

**Table 2:** Preparation of some oxazine derivatives by using SDS in water at room temperature

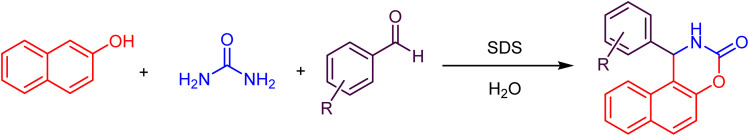								
Entry	Product	Thermal condition^a^		US condition^b^		*m*.p_obs._ (°C)	*m*.p_rep._ (°C)	Ref.
		Time (min)	Yield^c^ (%)	Time (min)	Yield^c^ (%)			
1	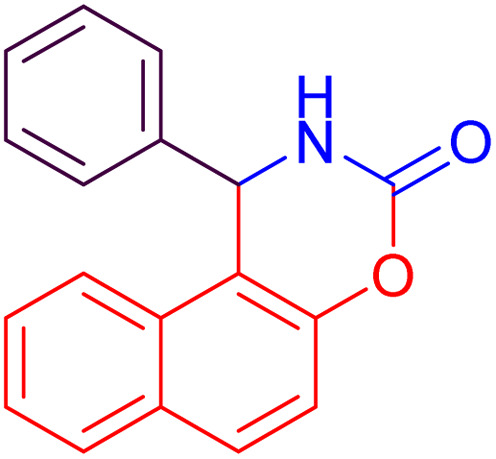	27	87	15	90	210–222	220–223	46
2	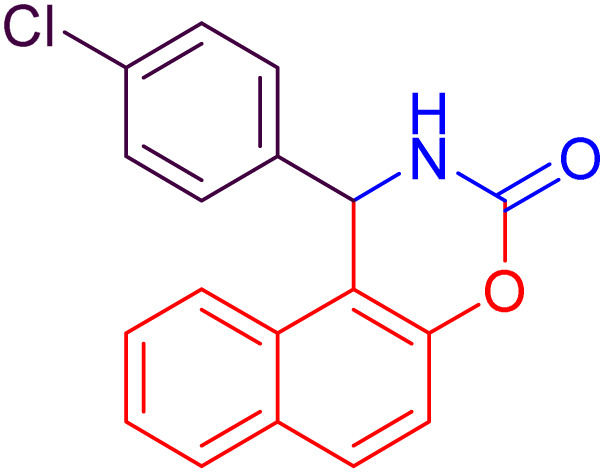	22	93	10	97	212–214	210–214	46
3	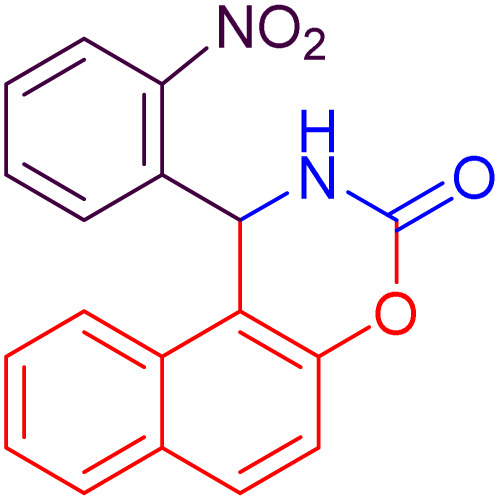	24	92	10	95	104–106	104–106	50
4	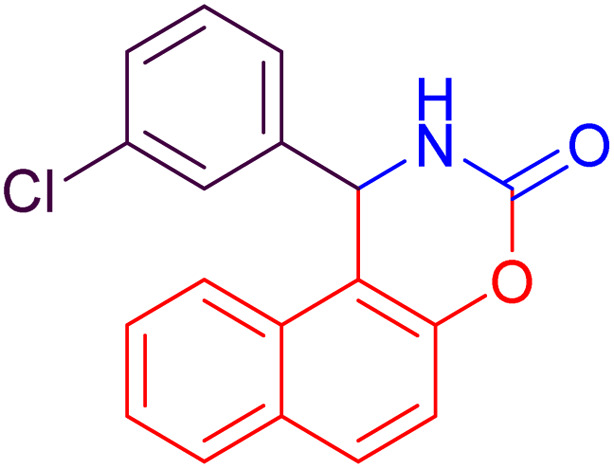	26	92	15	96	228–231	227–230	51
5	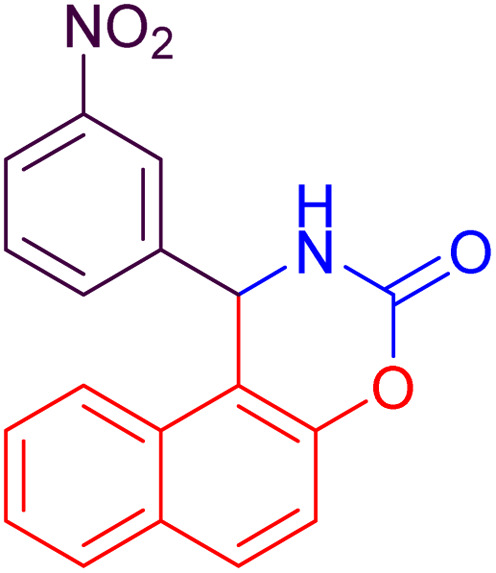	26	90	15	94	210–212	211–213	47
6	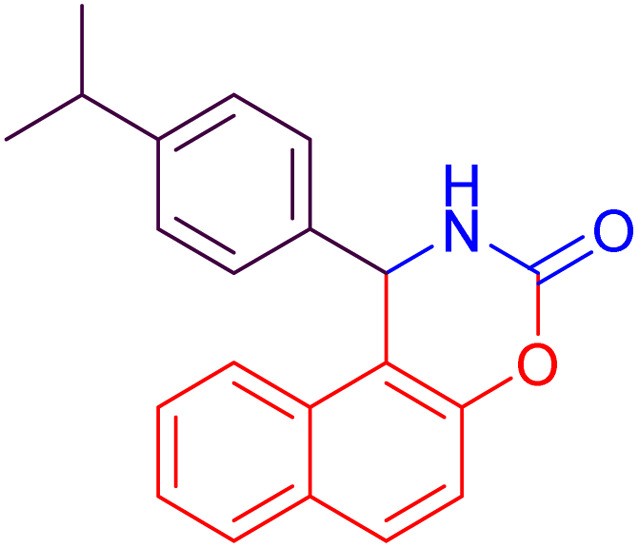	34	84	25	87	240–242	241–243	51
7	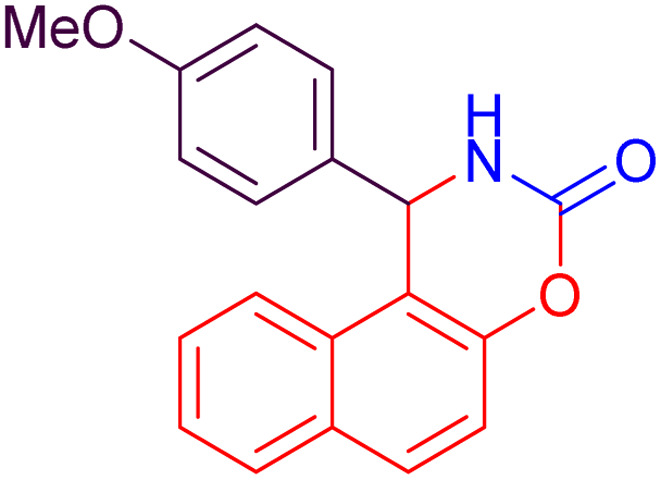	35	82	25	86	188–190	187–189	47
8	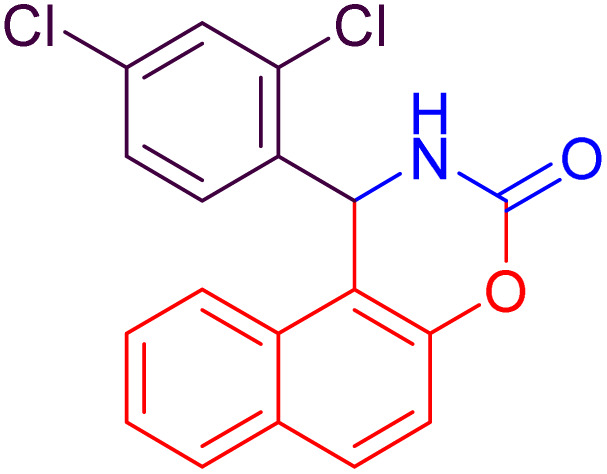	20	91	10	92	215–216	216–217	52
9	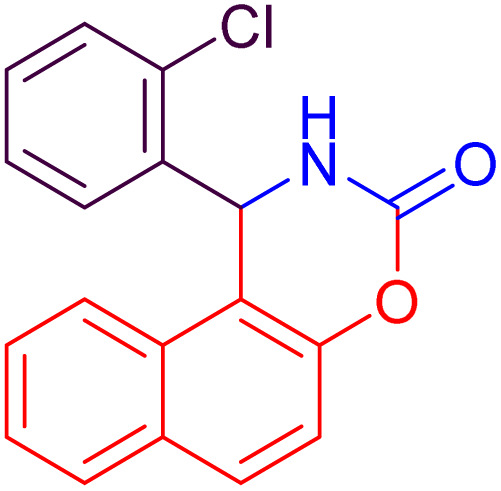	26	88	15	94	248–250	248–250	47
10	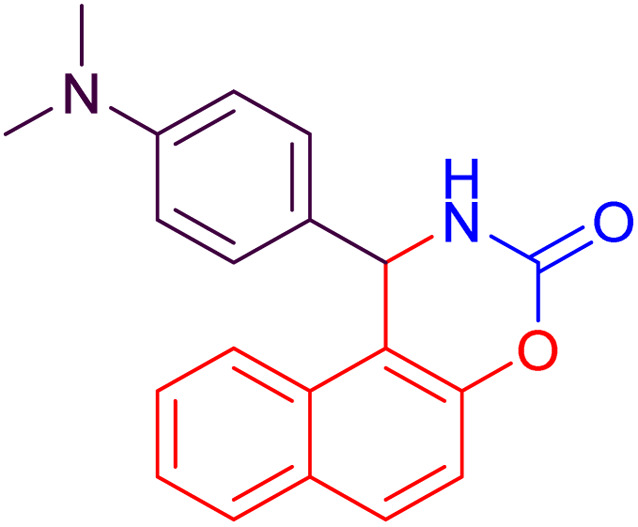	36	80	25	86	223–224	223–225	52
11	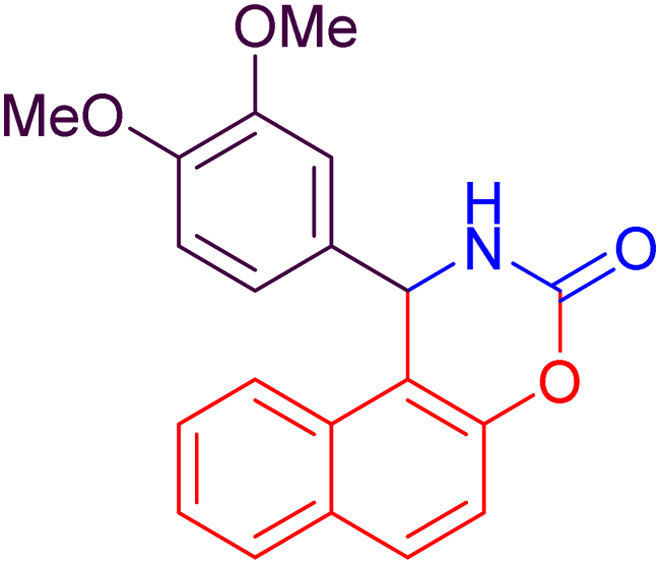	40	82	25	85	189–190	190–192	52
12	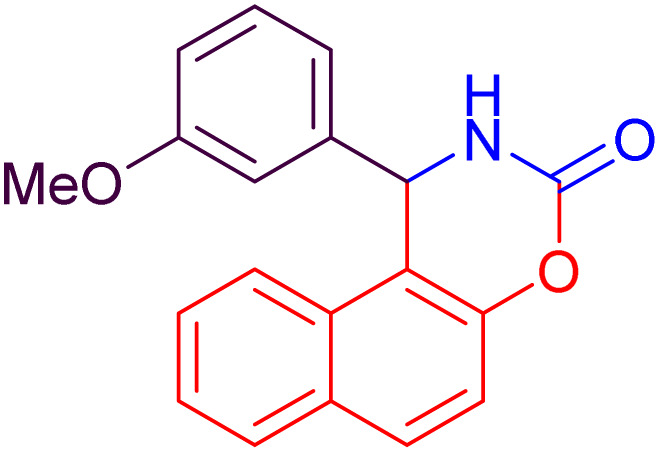	35	82	25	88	186–188	185–188	50

a Thermal conditions: β-naphthol (1 mmol), benzaldehyde (1 mmol), urea (1 mmol), H_2_O (5 mL), r.t, in the presence of 10% mol SDS.

b Ultrasound conditions: β-naphthol (1 mmol), benzaldehyde (1 mmol), urea (1 mmol), H_2_O (5 mL), power = 80 W, in the presence of 10% mol SDS.

c Isolate yields.

As stated in the reaction condition optimization section, the use of anionic surfactants such as sodium dodecyl sulfate (SDS) significantly increased the reaction yield, whereas cationic and nonionic surfactants exhibited lower yields. This difference is mainly attributed to the surface charge of the micelles and electrostatic effects. According to previous reports,^49^ the electrical double layer in anionic micelles leads to the accumulation of positive ions (including H^+^) near the micelle surface, creating a localized acidic environment that, similar to acidic catalysts, enhances the reaction rate. Therefore, SDS micelles not only increase the local concentration of reactants but also act as acidic catalysts. In contrast, cationic surfactants such as CTAB, with positively charged micelle surfaces, repel H^+^ ions and thus do not provide a favorable environment for reaction acceleration, while nonionic surfactants lack surface charge and consequently do not exhibit similar catalytic effects. Ultimately, the difference in surfactant performance is primarily due to the role of the electrical double layer and its influence on the local pH at the micelle surface, which aligns with previous studies and highlights the importance of surfactant selection in designing micellar-based reactions.

### 3.2. Reaction mechanism

In this multicomponent reaction, which is carried out in a micellar medium using the surfactant SDS, three key starting materials are involved: an aromatic aldehyde, β-naphthol, and urea. The micellar environment plays a crucial role in facilitating and enhancing the reaction's efficiency. Specifically, hydrophobic compounds such as the aldehyde and β-naphthol are concentrated within the hydrophobic core of the micelle, while urea, due to its polar nature, is mainly located in the aqueous phase or at the interface between the aqueous phase and the micellar core. This purposeful spatial arrangement leads to an increased effective concentration of reactants at the phase boundary, thereby significantly improving both the yield and the reaction rate.

**Scheme 3:**
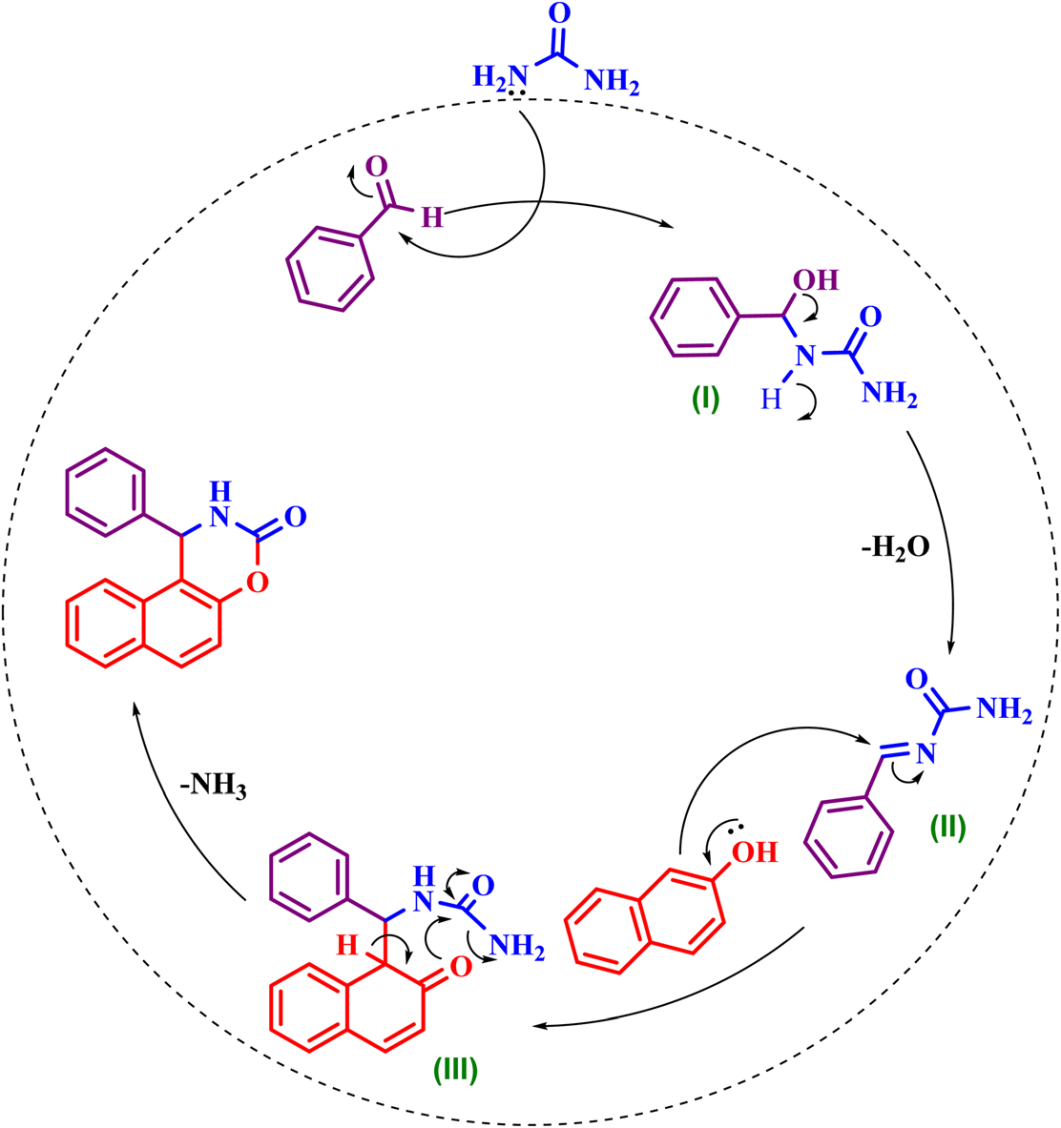
A plausible mechanism for the preparation of 1,2-dihydro-1-arylnaphtho [1,2-*e*]-[1,3]oxazine-3-ones in the presence of SDS.

As illustrated in Scheme 3, in the initial step of this reaction pathway, the carbonyl group of the aldehyde undergoes a condensation reaction with the amino group of urea, resulting in the formation of an acylimine intermediate and the elimination of a water molecule. This reactive intermediate provides a suitable platform for the subsequent steps of the reaction. In the next stage, β-naphthol, which is concentrated in the hydrophobic region of the micelle, acts as a potent nucleophile and attacks the acylimine intermediate, forming a new, more complex intermediate. Finally, as a result of the unique conditions provided by the micellar environment, the resulting intermediate undergoes an intramolecular cyclization process. In this step, an ammonia molecule is eliminated, and the final cyclic structure, known as naphthoxazinone, is formed.^53,54^

The high concentration of SDS (10 mol%) in water, well above its CMC (about 8 mM), ensures the formation of spherical micelles. The anionic head groups of SDS create a negatively charged surface that attracts H^+^ ions, making a proton-rich environment that acts as a “pseudo-acidic” catalyst, facilitating the protonation of aldehyde carbonyls and speeding up the formation of acylimine intermediates. The significant drop in yield at elevated temperatures (Table 1, entries 9–12) can be attributed not only to the increase in CMC, but also to the partial disruption of the electrical double layer and the decreased stability of the micellar structure at higher thermal energies. Furthermore, the superior performance of SDS compared to cationic (CTAB) and non-ionic surfactants (Triton X-100) quantitatively confirms that the electrostatic environment of the micellar interface is a key kinetic driver in this transformation.

### 3.3. Evaluating the effectiveness of this method compared to other approaches

Based on the data presented in Table 3, a comparison between previous methods and the current approach for the synthesis of naphthoxazinones clearly demonstrates the significant advantages of the method proposed in this study. As shown in the table, the reaction yield achieved by the present method is higher and more consistent than that of most previous protocols. Moreover, the reaction time is dramatically reduced when using SDS as a catalyst in combination with ultrasound irradiation, with completion achieved in only 10 minutes; even under ambient conditions, the reaction is completed within 23 minutes, whereas many previous methods require considerably longer reaction times. From the perspective of reaction conditions, the use of water as a solvent and SDS as a catalyst renders this process green, safe, and environmentally friendly. In contrast, a substantial portion of earlier methods rely on organic solvents, elevated temperatures, or expensive and metallic catalysts, which not only increase costs and environmental risks but also reduce operational simplicity. Additionally, the amount of catalyst required in the present method is lower than that of some comparable protocols. Overall, considering the data in the table, the present method by offering high yields, short reaction times, green conditions, low catalyst loading, and operational simplicity emerges as a superior and recommendable approach for the synthesis of naphthoxazinones.

**Table 3:** Evaluation of the catalytic efficiency of SDS relative to various catalysts documented in the literature^a^

Entry	Catalyst	Condition	Yield (%)	Time (min)	Ref.
1	[PyPS][HSO_4_] (15 mol%)	Solvent free, 150 °C	94	30	39
2	TMSCl (1.5 equiv.)	NaI (1.5 equiv.), r.t	79	108	40
3	Copper NPs (1 mg)	K_2_CO_3_, PEG-400, r.t, air atmosphere	96	60	41
4	HClO_4_/SiO_2_ (2 mol%)	Solvent free, 150 °C	88	60	42
5	ZnO-NPs (0.3 equiv)	Solvent free, r.t	92	40	46
6	Ompg-C_3_N_4_/SO_3_H (20 mg)	Solvent free, 100 °C	95	5	47
7	I_2_ (0.3 mmol)	Solvent free, 140–150 °C	93	5	51
8	FePO_4_ (20 mol%)	Solvent free, 150 °C	95	100	52
9	I_2_ (0.25 g)	Solvent free, 80 °C	92	5	53
10	Silica-bonded propyl-*S*-sulfonic acid 0.02 (0.68 mol%)	Solvent free, 150 °C	92	50	55
11	SDS (10 mol%)	H_2_O, US (80 W)	97	10	This work
12	SDS (10 mol%)	H_2_O, r.t	95	23	This work

a Reaction conditions: 4-chlorobenzaldehyde (1.0 mmol), urea (1.0 mmol) and β-naphthol (1.0 mmol).

## 4 Conclusion

In this study, an efficient and environmentally benign protocol for the synthesis of biologically important naphthoxazinone derivatives was successfully developed *via* a three-component reaction of β-naphthol, aryl aldehydes, and urea using sodium dodecyl sulfate (SDS) as a micellar catalyst in aqueous medium. The reaction was performed under both room temperature and ultrasonic irradiation conditions, with the latter significantly enhancing reaction rates and product yields (up to 97% within 10 minutes) while maintaining operational simplicity. The catalytic efficiency of SDS originates from its dual role: creating a hydrophobic microenvironment that concentrates nonpolar reactants within the micelle core, while its anionic head groups generate an electrical double layer at the micelle–water interface, providing localized “pseudo-acidic” conditions that facilitate the condensation and cyclization steps. This synergistic effect leads to increased local concentration of reactants and lower activation energy. The method offers several advantages, including the use of water as a safe and accessible solvent, mild reaction conditions, low catalyst loading, short reaction times, high yields, and broad substrate tolerance for various aryl aldehydes. Furthermore, the catalyst is inexpensive, commercially available, and requires no special handling or preparation. In summary, the combination of micellar catalysis with ultrasonic irradiation provides a simple, rapid, sustainable, and highly efficient approach for the synthesis of naphthoxazinone derivatives, offering a promising alternative to conventional methods that often require toxic solvents, elevated temperatures, or expensive catalysts.

## Author contributions

first author: investigation, formal analysis, writing – original draft. last author: conceptualization, supervision, writing – review & editing, project administration.

## Conflicts of interest

There are no conflicts to declare.

## Supplementary Material

RA-016-D5RA08594B-s001

## Data Availability

The data supporting this article have been included as part of the supplementary information (SI). Supplementary information is available. See DOI: https://doi.org/10.1039/d5ra08594b.
