# Supplementary material for: Micellar effects upon synthesis of 1,2-dihydro-1-arylnaphtho[1,2-e][1,3]oxazine-3-ones in water at room temperature
Source: RSC Adv. 2026 May 19;16(29):26746–55. doi: 10.1039/d5ra08594b (PMC13187904; doi:10.1039/d5ra08594b)
Supplement: RA-016-D5RA08594B-s001 [file RA-016-D5RA08594B-s001.pdf]

Supplementary Information (SI) for RSC Advances.
This journal is © The Royal Society of Chemistry 2026

Supporting information

Micellar effects upon synthesis of 1,2-Dihydro-1-arylnaphtho[1,2-e]-[1,3]oxazine-3-ones in water at room temperature

Saeedeh Asadian, Mohsen Moradian*, Javad Safari

Department of Organic Chemistry, Faculty of Chemistry, University of Kashan, Kashan, P.O. Box 87317-51167, I. R.Iran

FT-IR and ^{1}H NMR spectra of synthesized compounds:

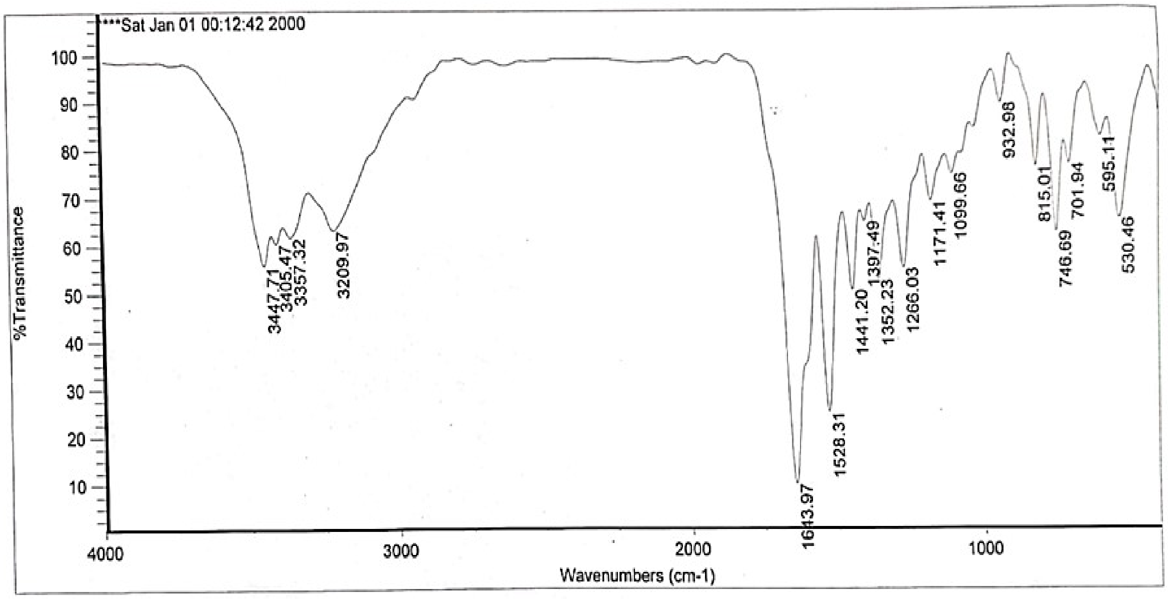

Figure 1: FT-IR spectrum of 1-Phenyl-1,2-dihydro-naphtho[1,2-e][1,3]oxazin-3-one **(4a)** in KBr.

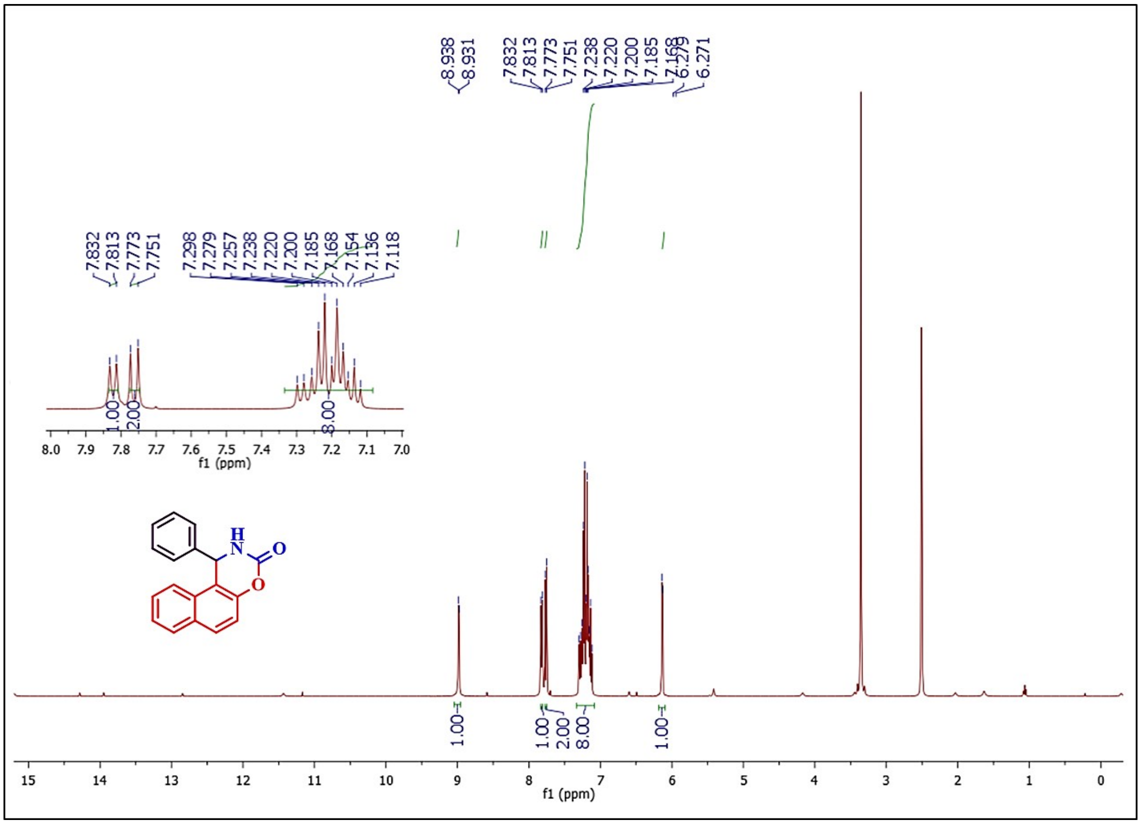

Figure 2: ^{1}H NMR spectrum (400 MHz) of 1-Phenyl-1,2-dihydro-naphtho[1,2-e][1,3]oxazin-3-one **(4a)** in DMSO.

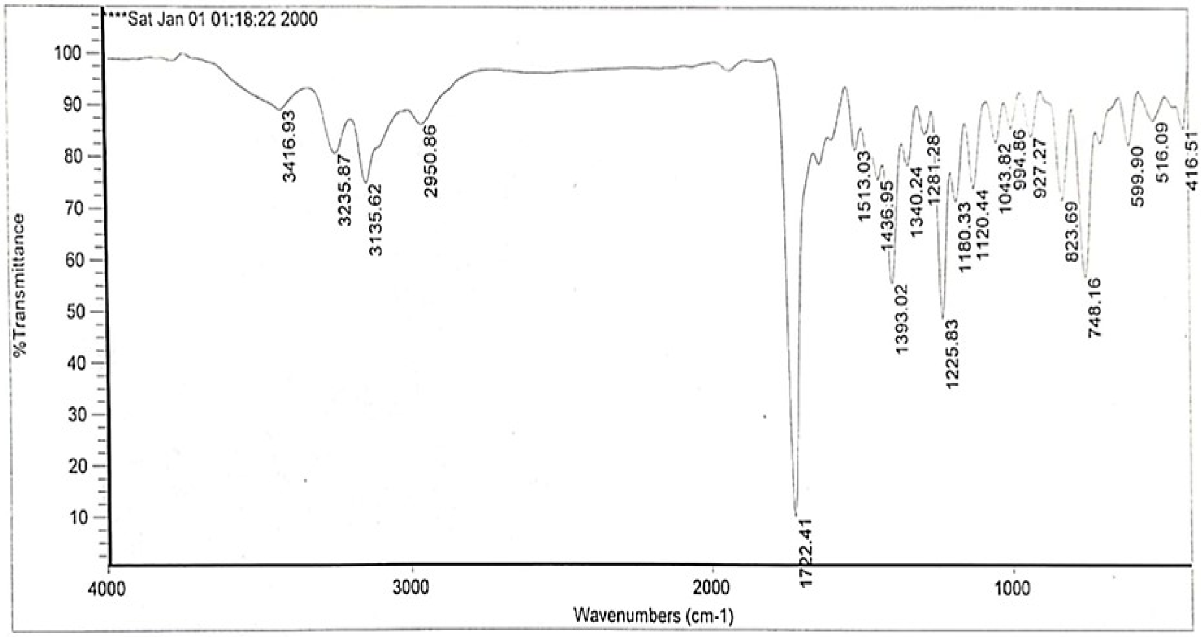

Figure 3: FT-IR spectrum 1-(4-Chlorophenyl)-1,2-dihydro-naphtho[1,2-e][1,3]oxazin-3-one **(4b)** in KBr.

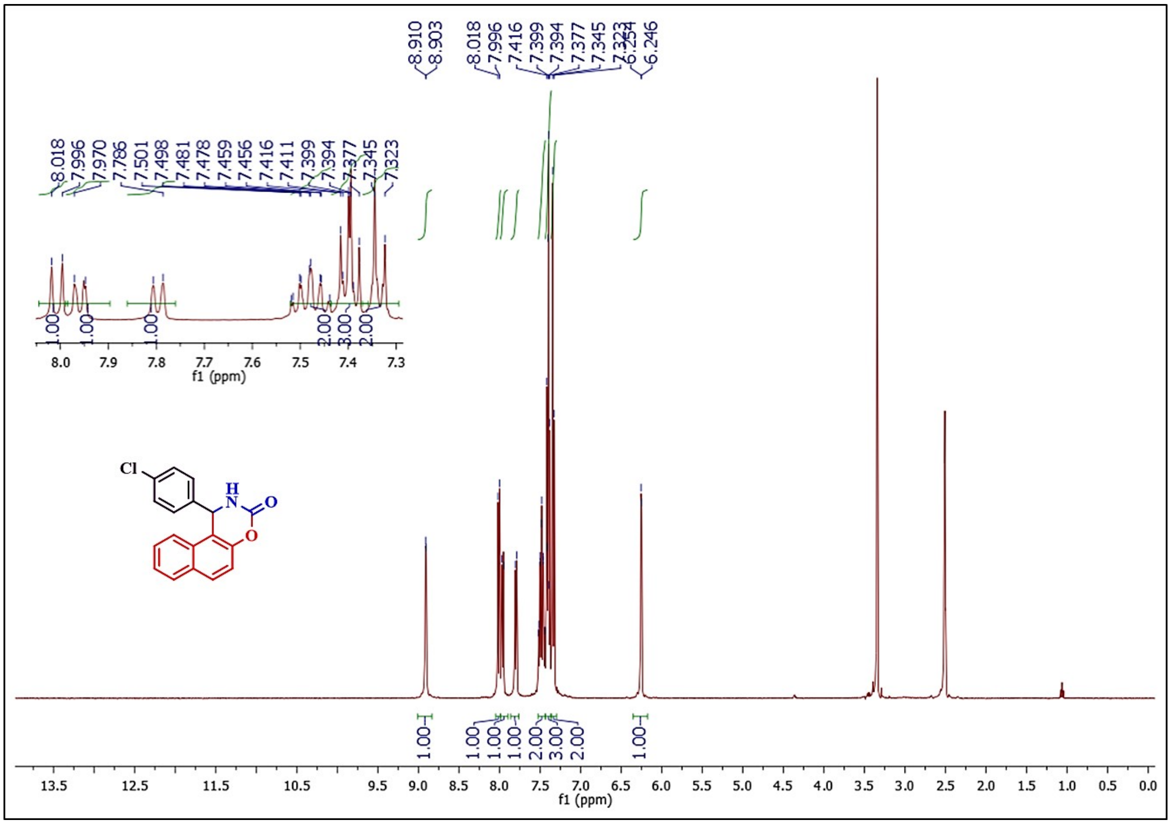

Figure 4: ^{1}H NMR spectrum (400 MHz) of 1-(4-Chlorophenyl)-1,2-dihydro-naphtho[1,2-e][1,3]oxazin-3-one **(4b)** in DMSO.

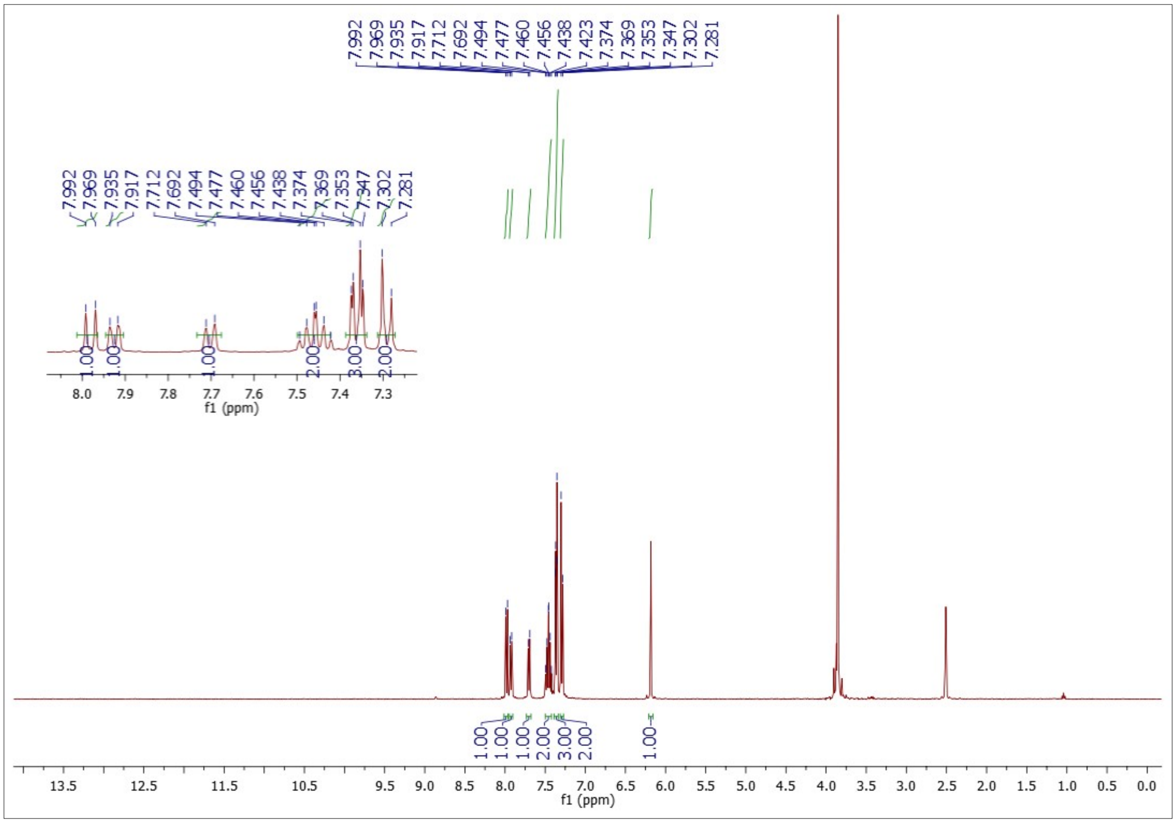

Figure 5: ^{1}H NMR spectrum (400 MHz) of 1-(4-Chlorophenyl)-1,2-dihydro-naphtho[1,2-e][1,3]oxazin-3-one **(4b)** in D_2O.

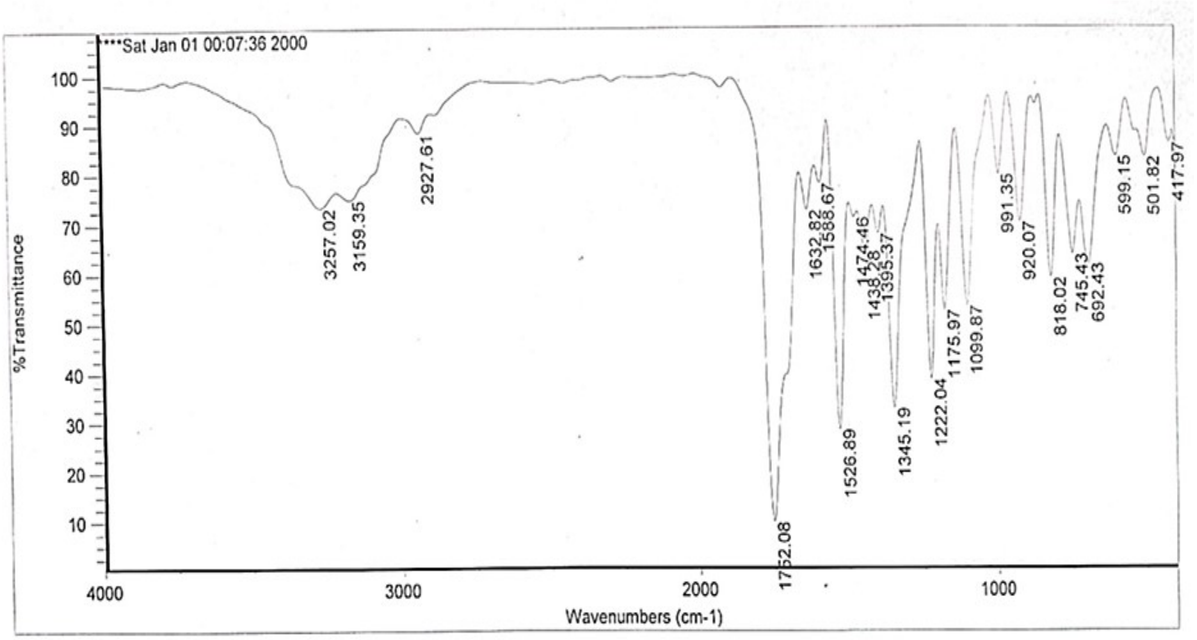

Figure 6: FT-IR spectrum of 1-(2-Nitrophenyl)-1,2-dihydro-naphtho[1,2-e][1,3]oxazin-3-one **(4c)** in KBr.

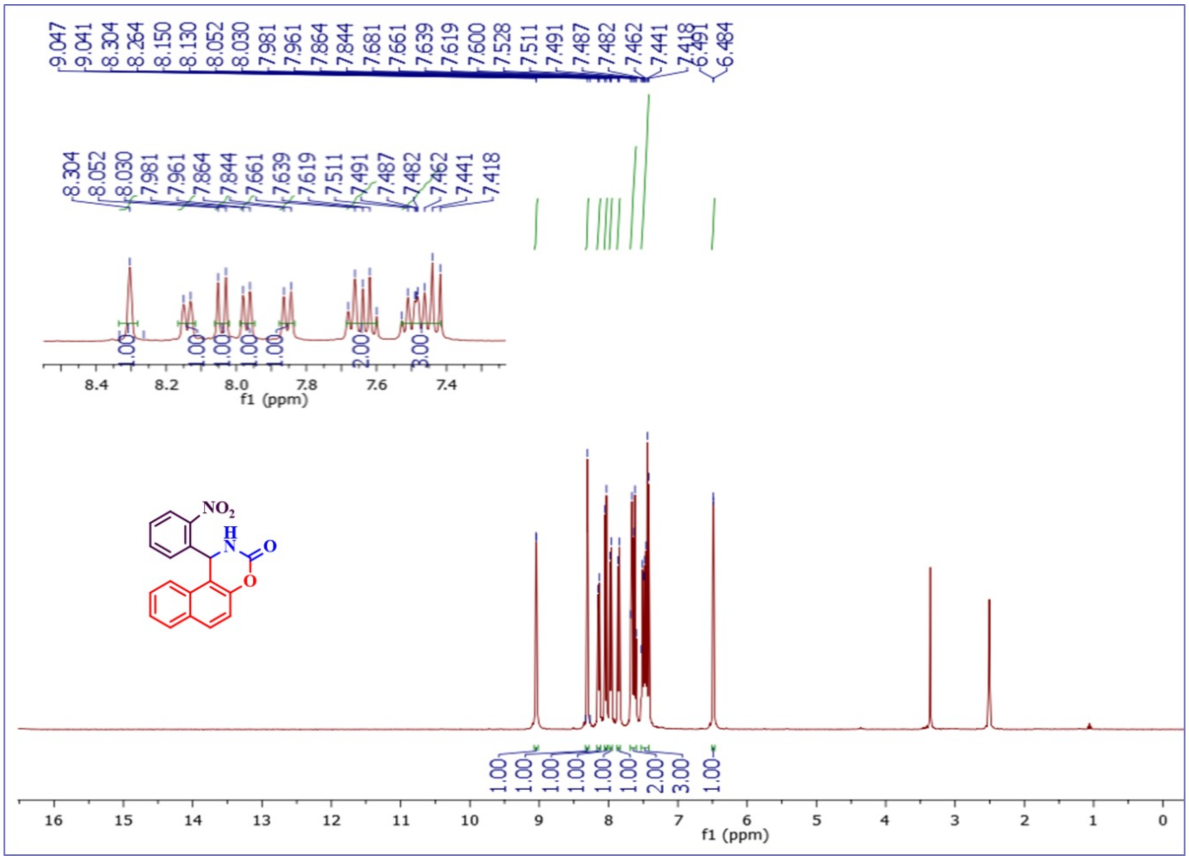

Figure 7: ^{1}H NMR spectrum (400 MHz) of 1-(2-Nitrophenyl)-1,2-dihydro-naphtho[1,2-e][1,3]oxazin-3-one **(4c)** in DMSO.

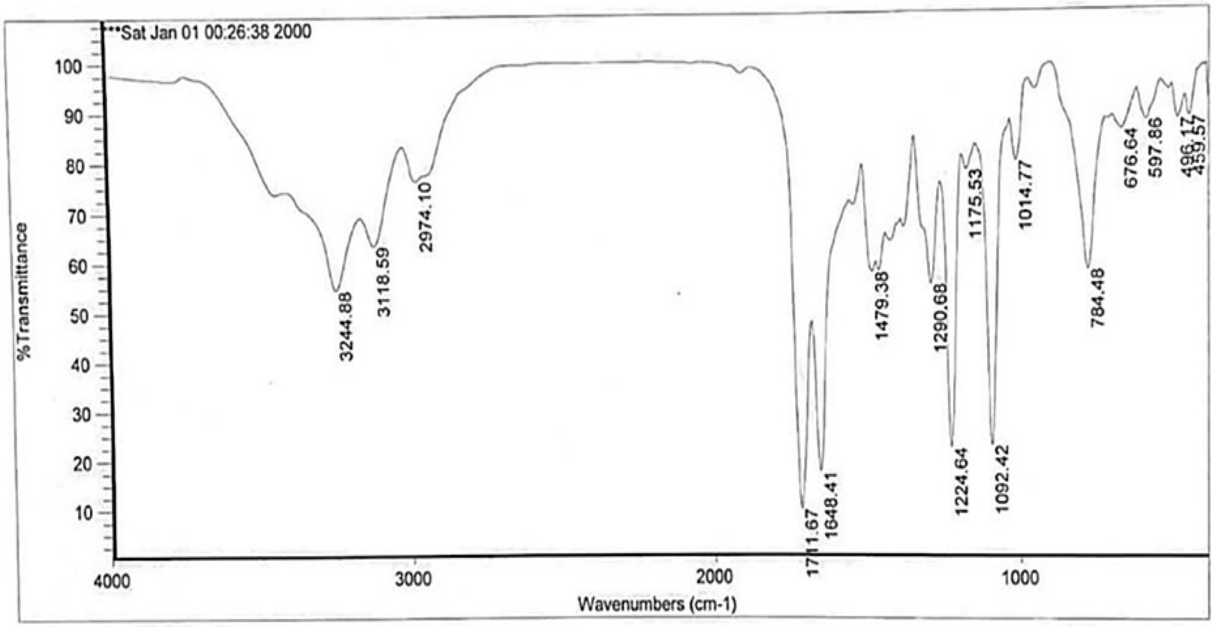

Figure 8: FT-IR spectrum of 1-(3-Chlorophenyl)-1,2-dihydro-naphtho[1,2-e][1,3]oxazin-3-one **(4d)** in KBr.

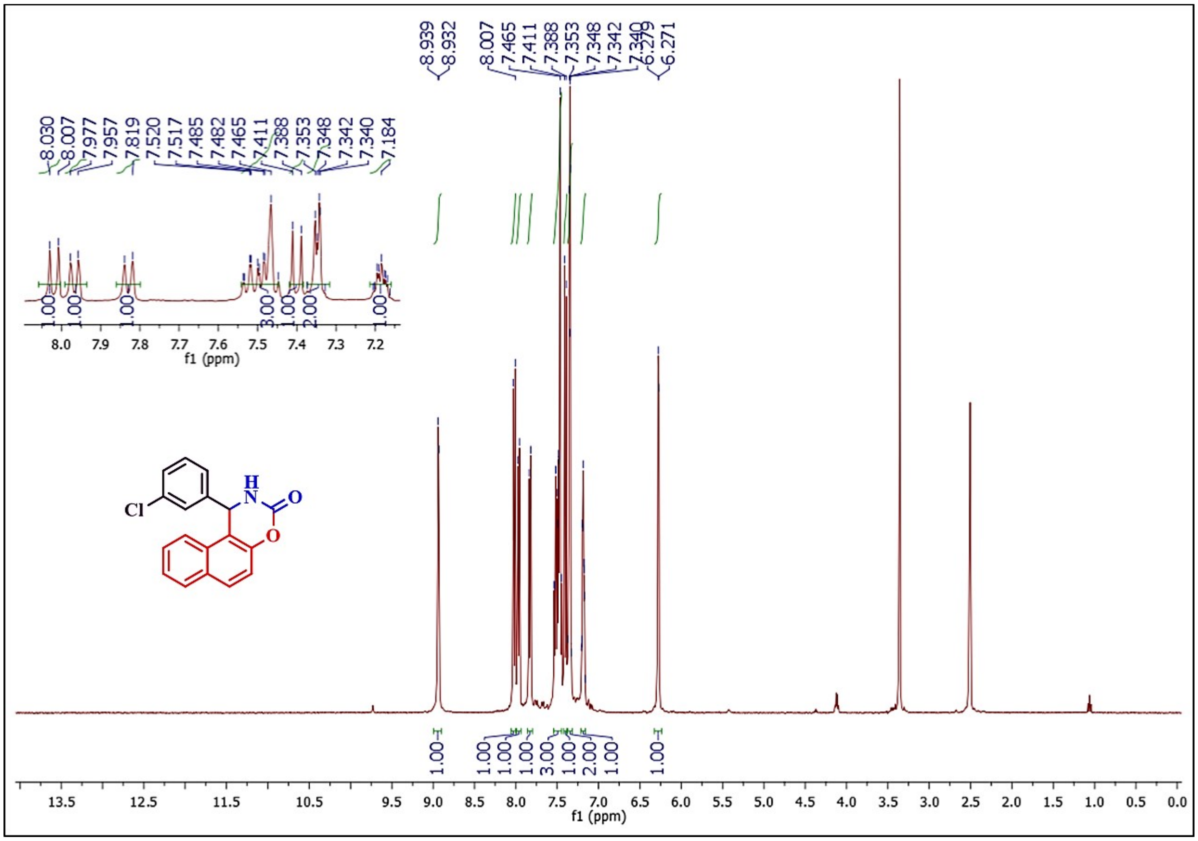

Figure 9: ^{1}H NMR spectrum (400 MHz) of 1-(3-Chlorophenyl)-1,2-dihydro-naphtho[1,2-e][1,3]oxazin-3-one **(4d)** in DMSO.

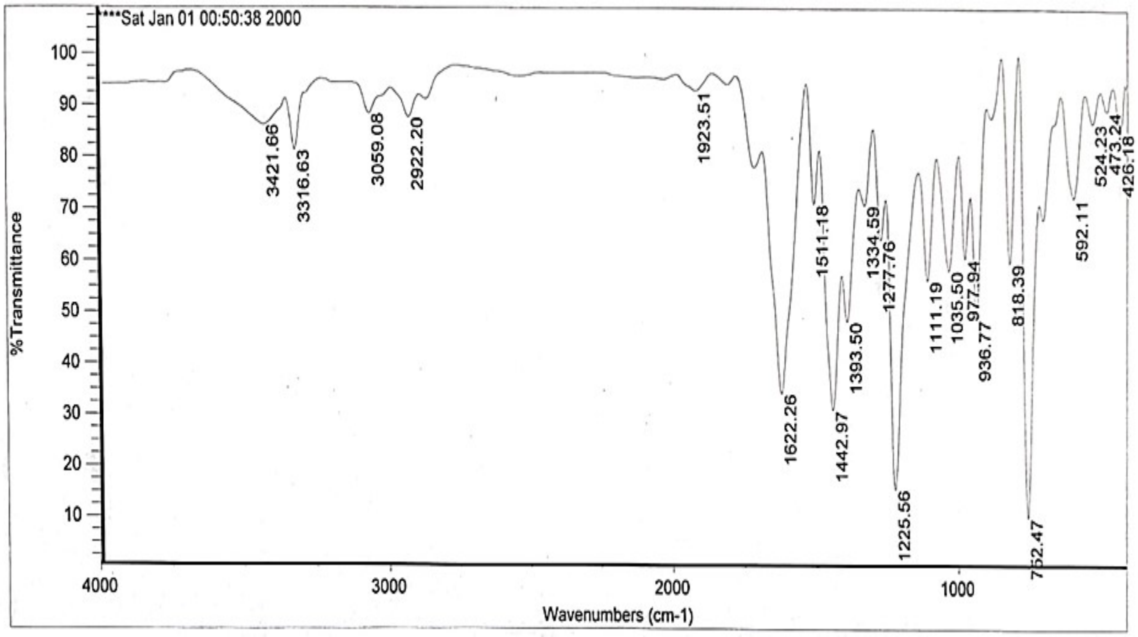

Figure 10: FT-IR spectrum of 1-(3-nitrophenyl)-1,2-dihydronaphtho[1,2-e][1,3]oxazin-3-one. **(4e)** in KBr.

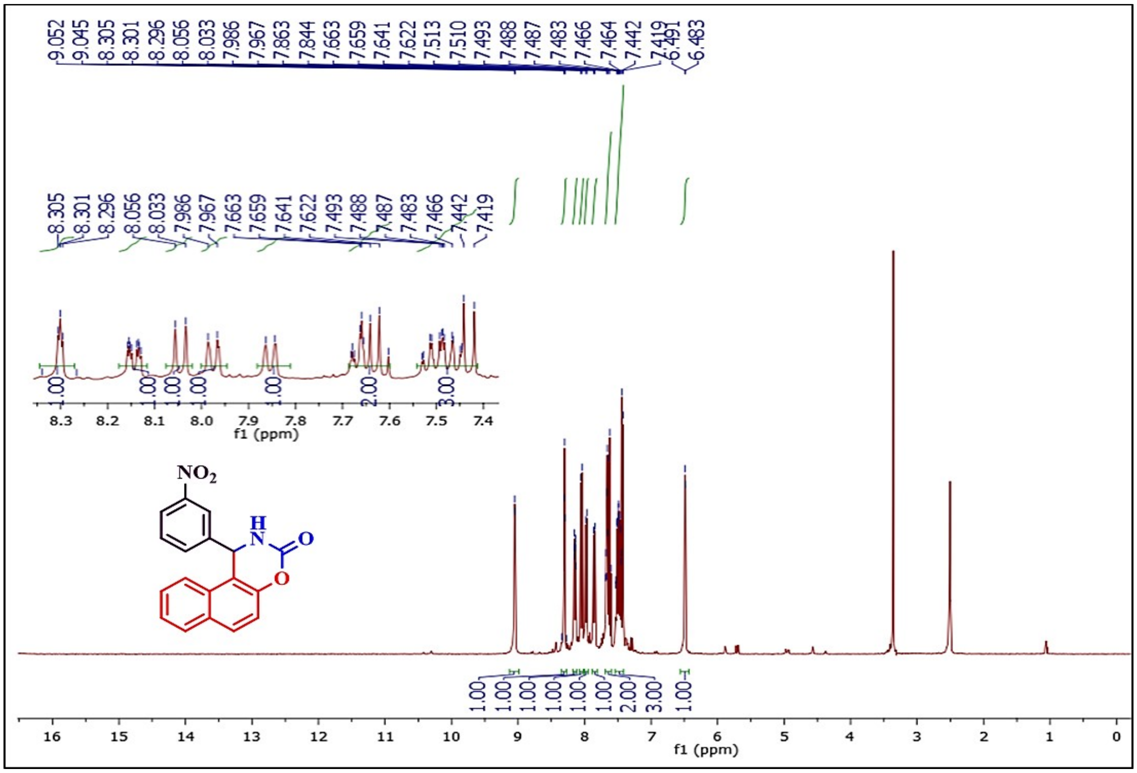

Figure 11: ^{1}H NMR spectrum (400 MHz) of 1-(3-nitrophenyl)-1,2-dihydronaphtho[1,2-e][1,3]oxazin-3-one **(4e)** in DMSO.

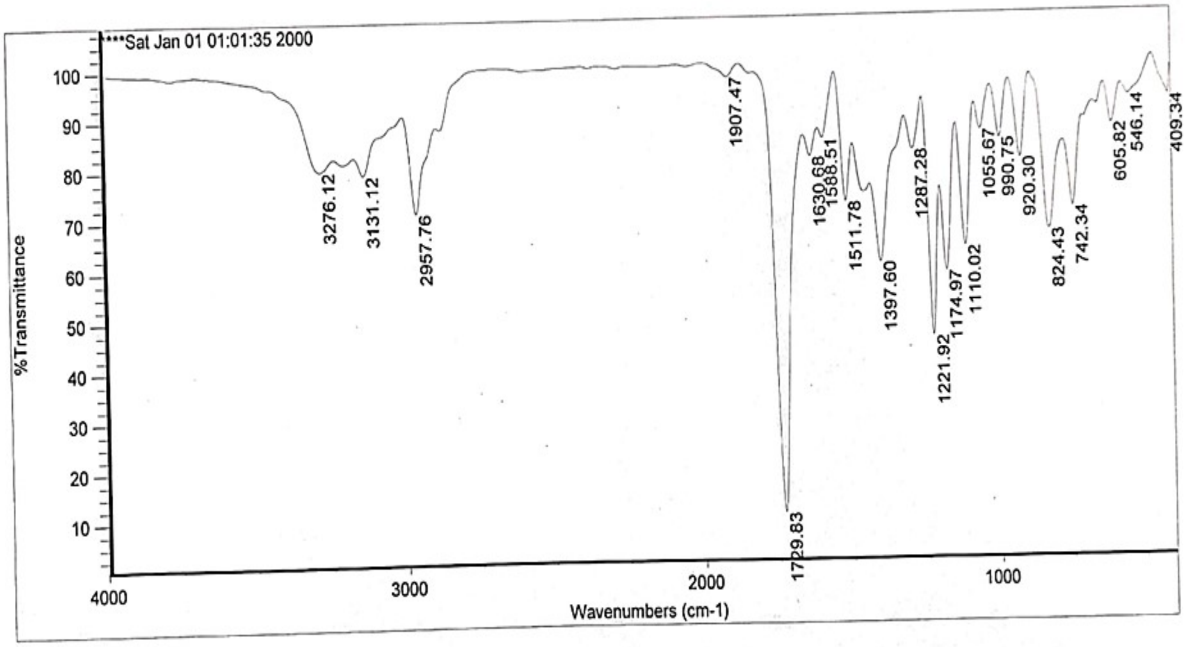

Figure 12: FT-IR spectrum of 1-(4-Iso-propylphenyl)-1,2-dihydro-naphtho[1,2-e][1,3]oxazin-3-one **(4f)** in KBr.

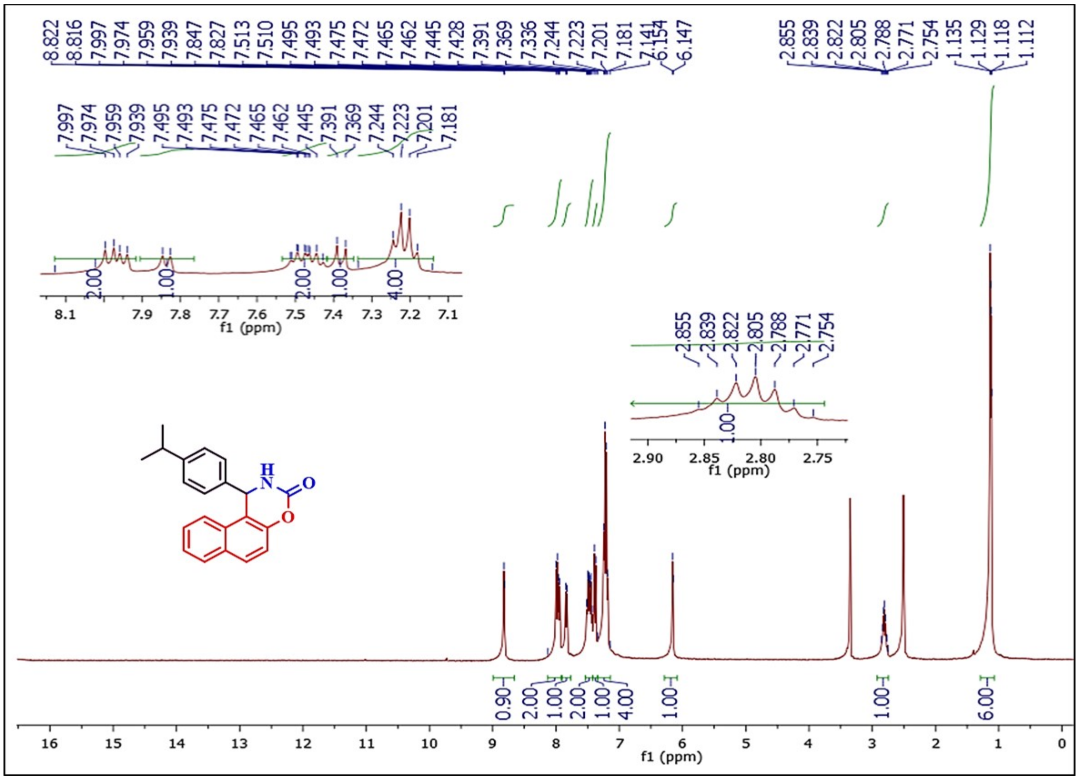

Figure 13: ^{1}H NMR spectrum (400 MHz) of 1-(4-Iso-propylphenyl)-1,2-dihydro-naphtho[1,2-e][1,3]oxazin-3-one **(4f)** in DMSO.

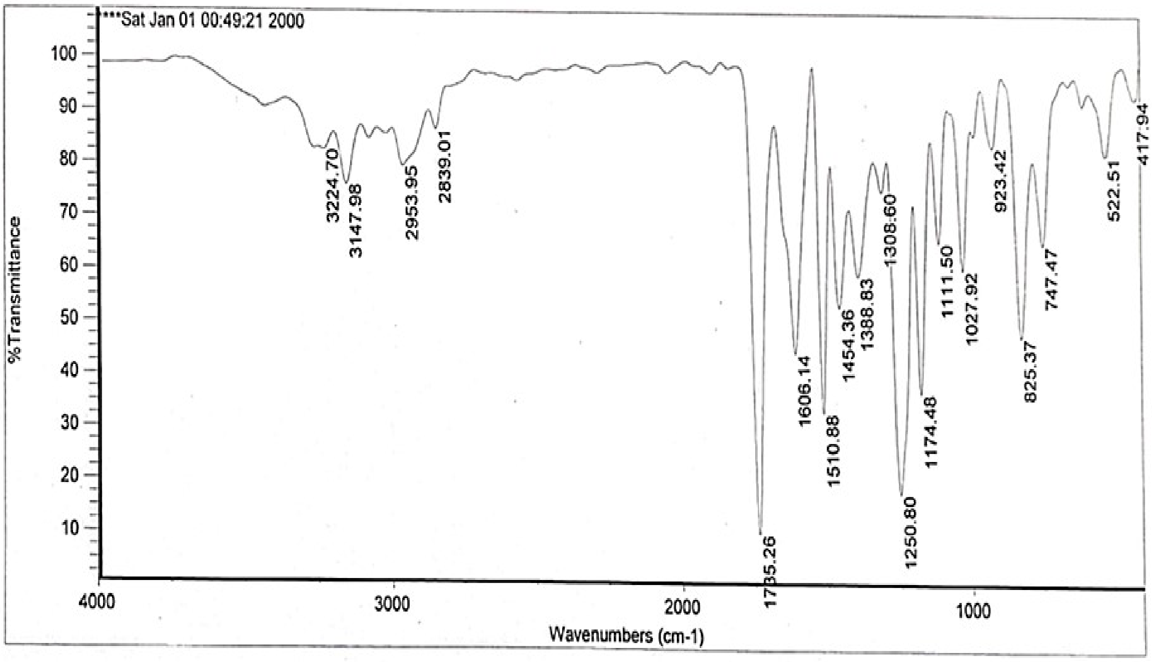

Figure 14: FT-IR spectrum of 1-(4-Methoxyphenyl)-1,2-dihydro-naphtho[1,2-e][1,3] oxazin-3-one **(4g)** in KBr.

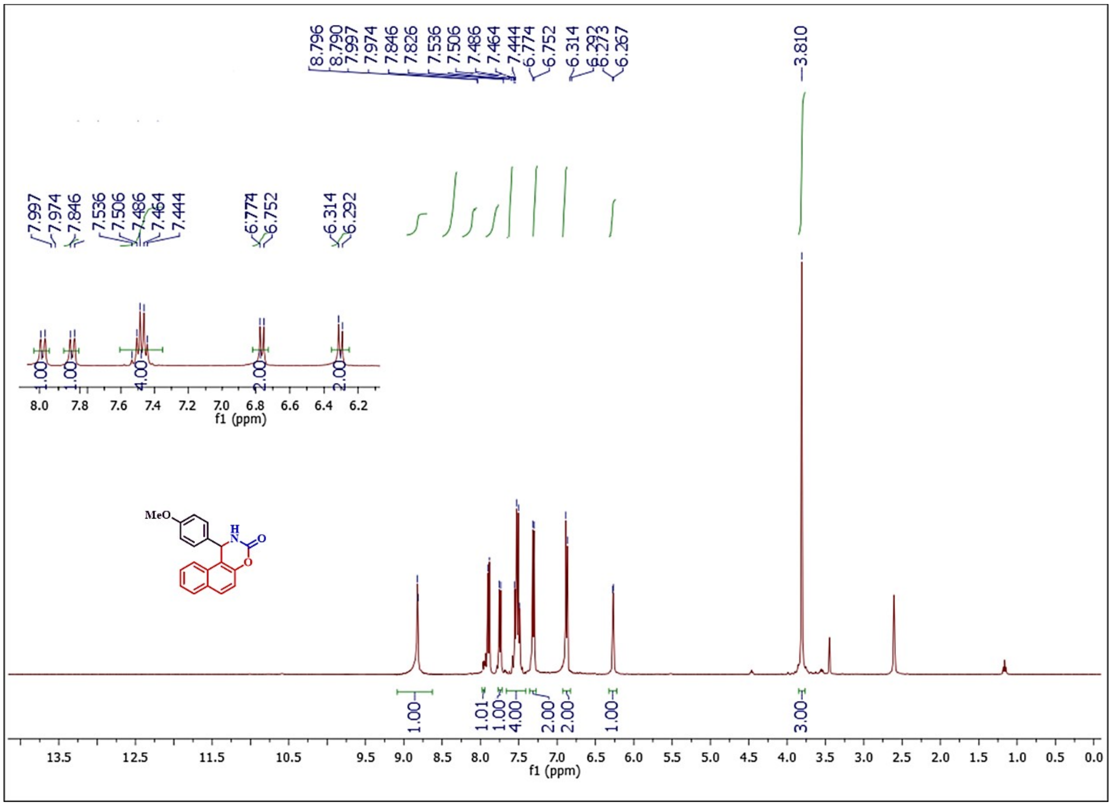

Figure 15: ^{1}H NMR spectrum (400 MHz) of 1-(4-Methoxyphenyl)-1,2-dihydro-naphtho[1,2-e][1,3] oxazin-3-one **(4g)** in DMSO.

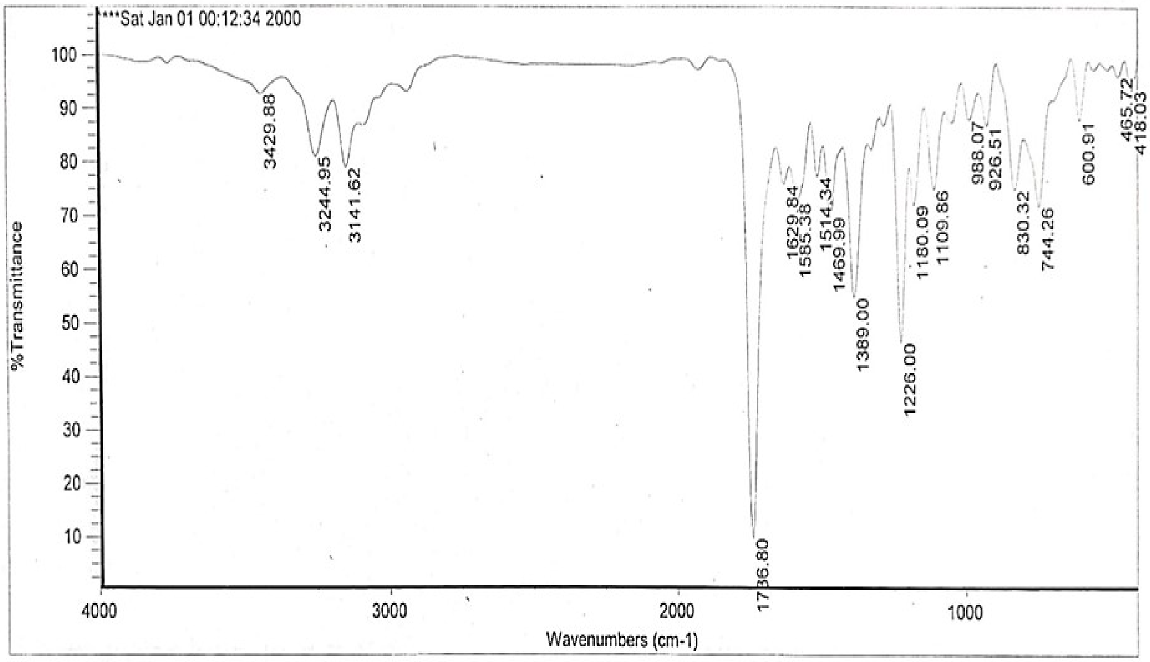

Figure 16: FT-IR spectrum of 1-(2,4-Dichlorophenyl)-1,2-dihydronaphtho[1,2-e][1,3]oxazin-3-one **(4h)** in KBr.

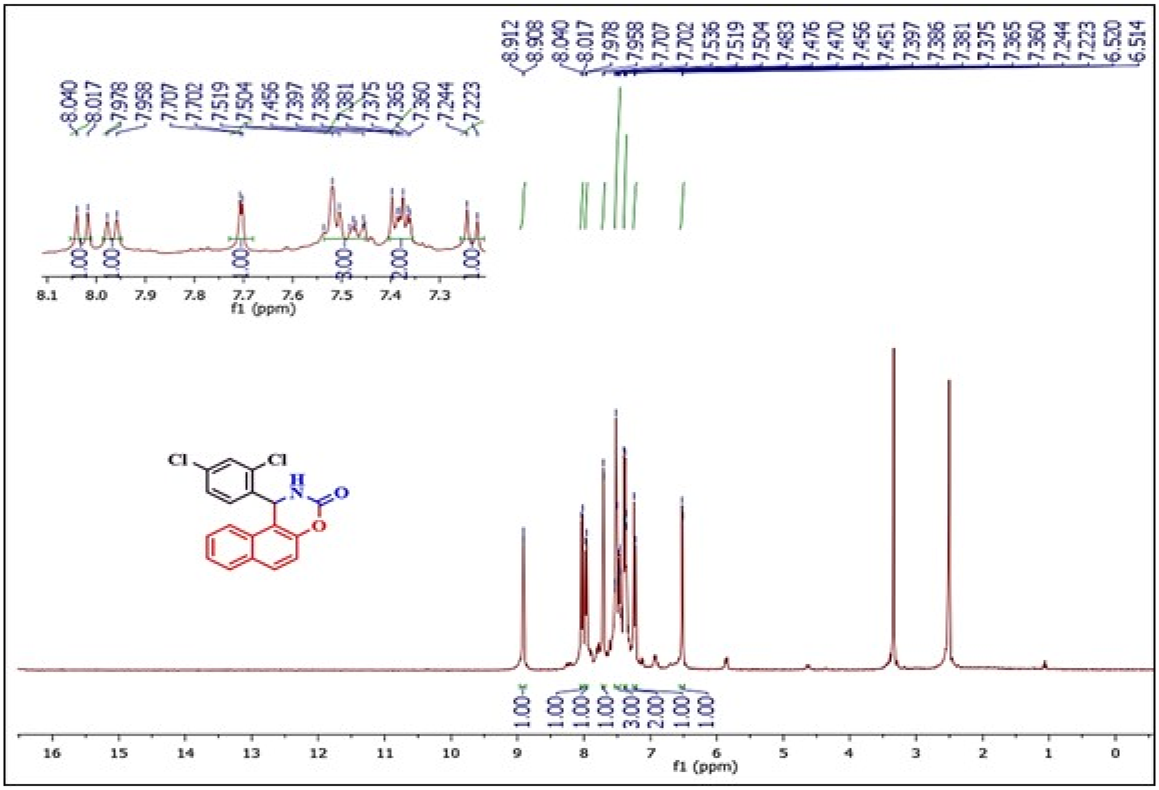

Figure 17: ^{1}H NMR spectrum (400 MHz) of 1-(2,4-Dichlorophenyl)-1,2-dihydronaphtho[1,2-e][1,3]oxazin-3-one **(4h)** in DMSO.

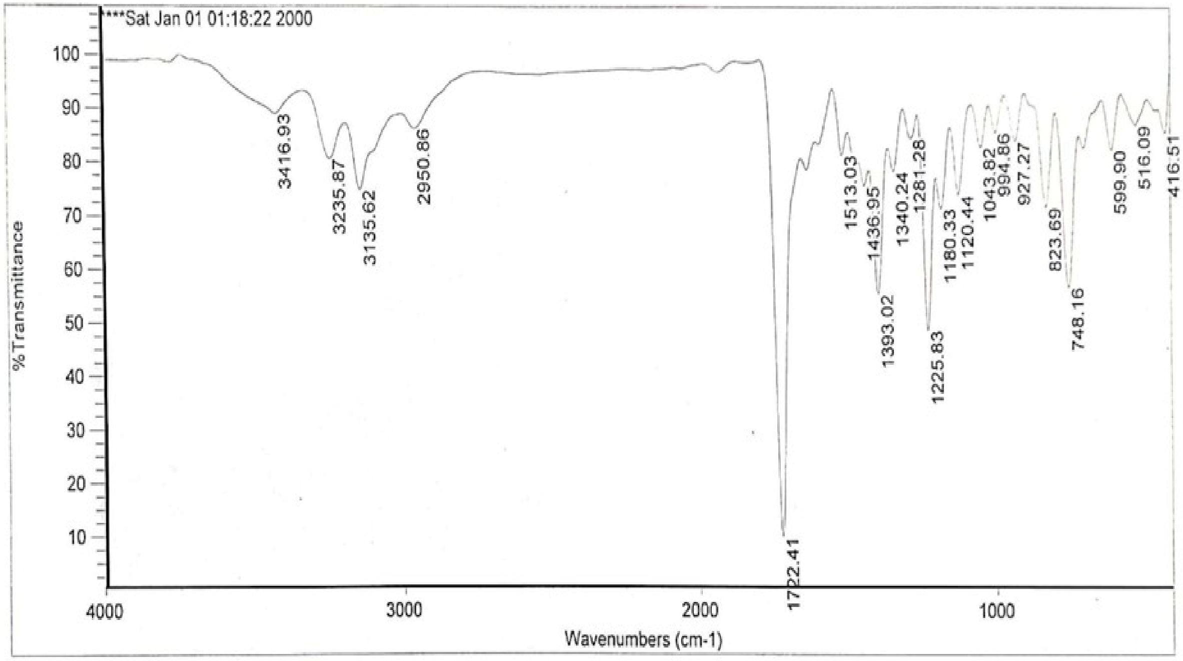

Figure 18: FT-IR spectrum of 1-(2-Chlorophenyl)-1,2-dihydronaphtho [1,2-e][1,3] oxazin-3-one **(4i)** in KBr.

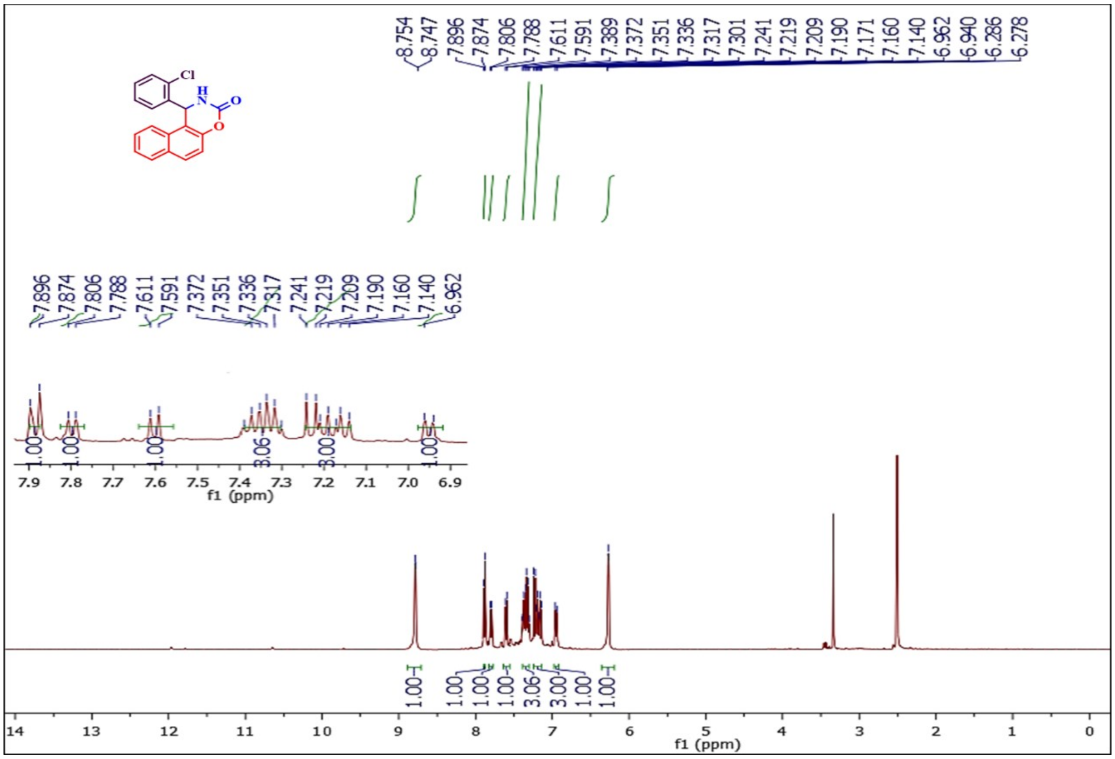

Figure 19: ^{1}H NMR spectrum (400 MHz) of 1-(2-Chlorophenyl)-1,2-dihydronaphtho [1,2-e][1,3] oxazin-3-one **(4i)** in DMSO.

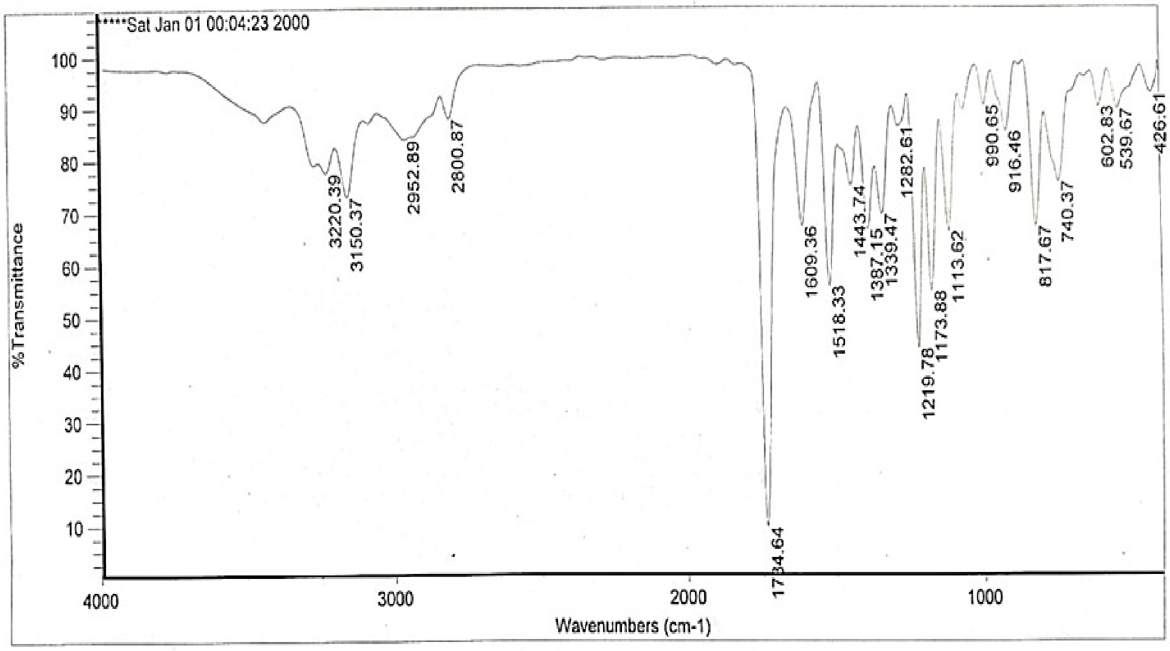

Figure 20: FT-IR spectrum of 1-(4-dimethylaminophenyl)-1,2-dihydronaphtho[1,2-e][1,3]oxazin-3-one **(4j)** in KBr.

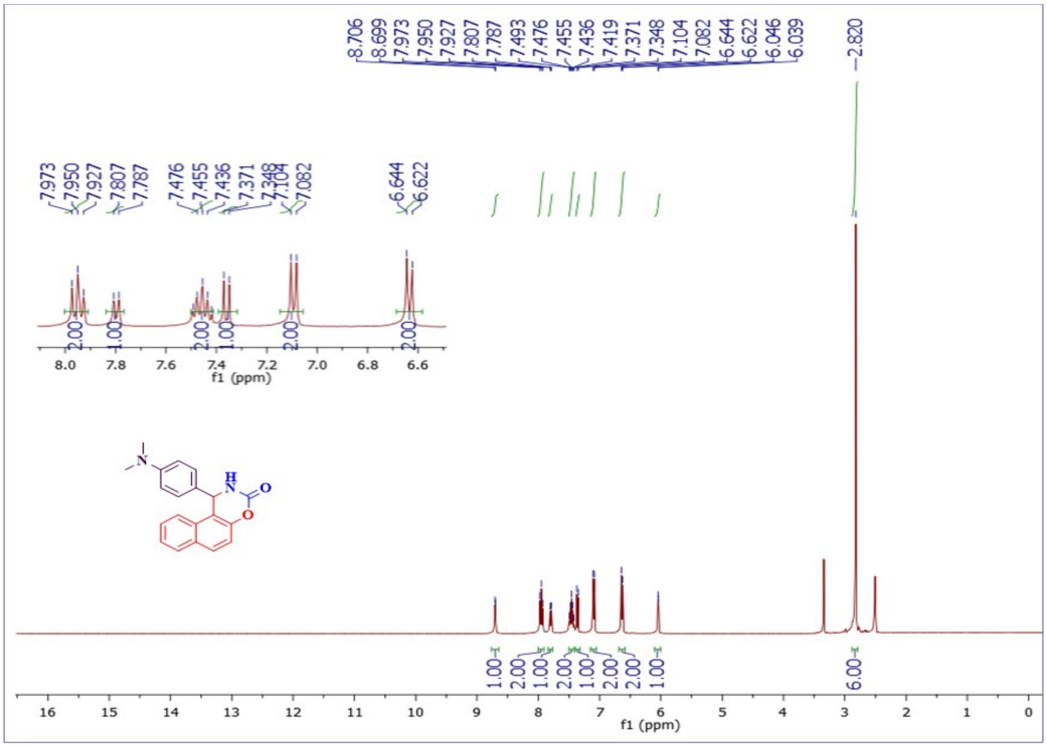

Figure 21: ^{1}H NMR spectrum (400 MHz) of 1-(4-dimethylaminophenyl)-1,2-dihydronaphtho[1,2-e][1,3]oxazin-3-one **(4j)** in DMSO.

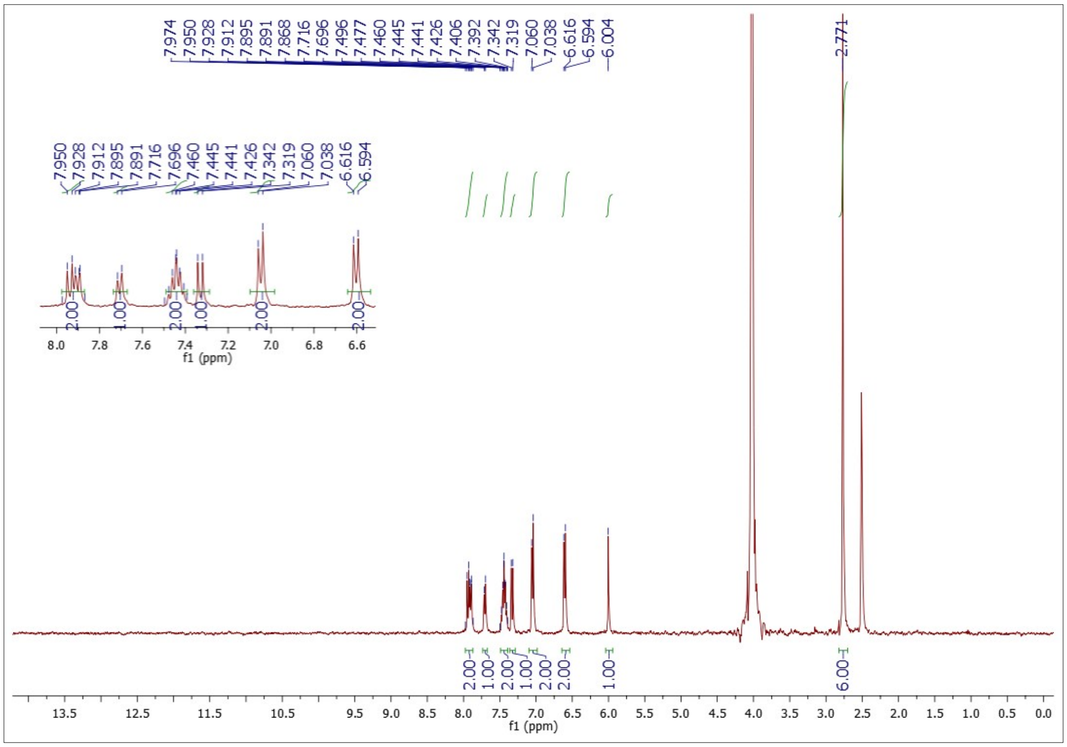

Figure 22: ^{1}H NMR spectrum (400 MHz) of 1-(4-dimethylaminophenyl)-1,2-dihydronaphtho[1,2-e][1,3]oxazin-3-one **(4j)** in D_2O.

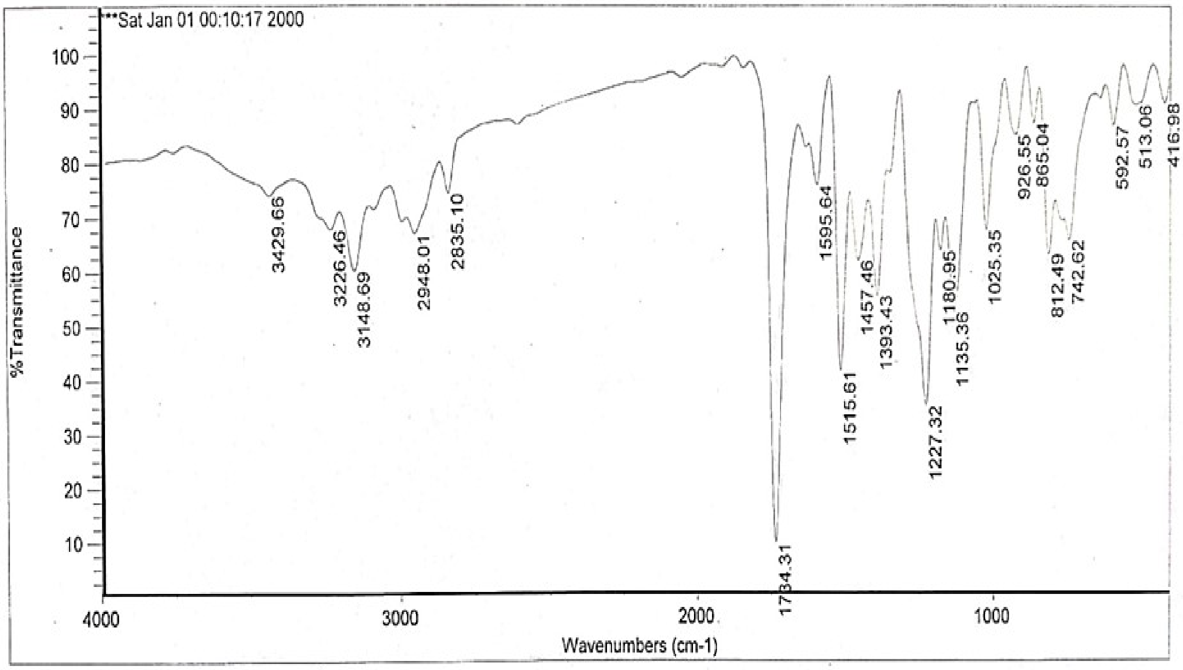

Figure 23: FT-IR spectrum of 1-(3,4-Dimethoxyphenyl)-1,2-dihydro-naphtho[1,2-e][1,3]oxazin-3-one **(4k)** in KBr.

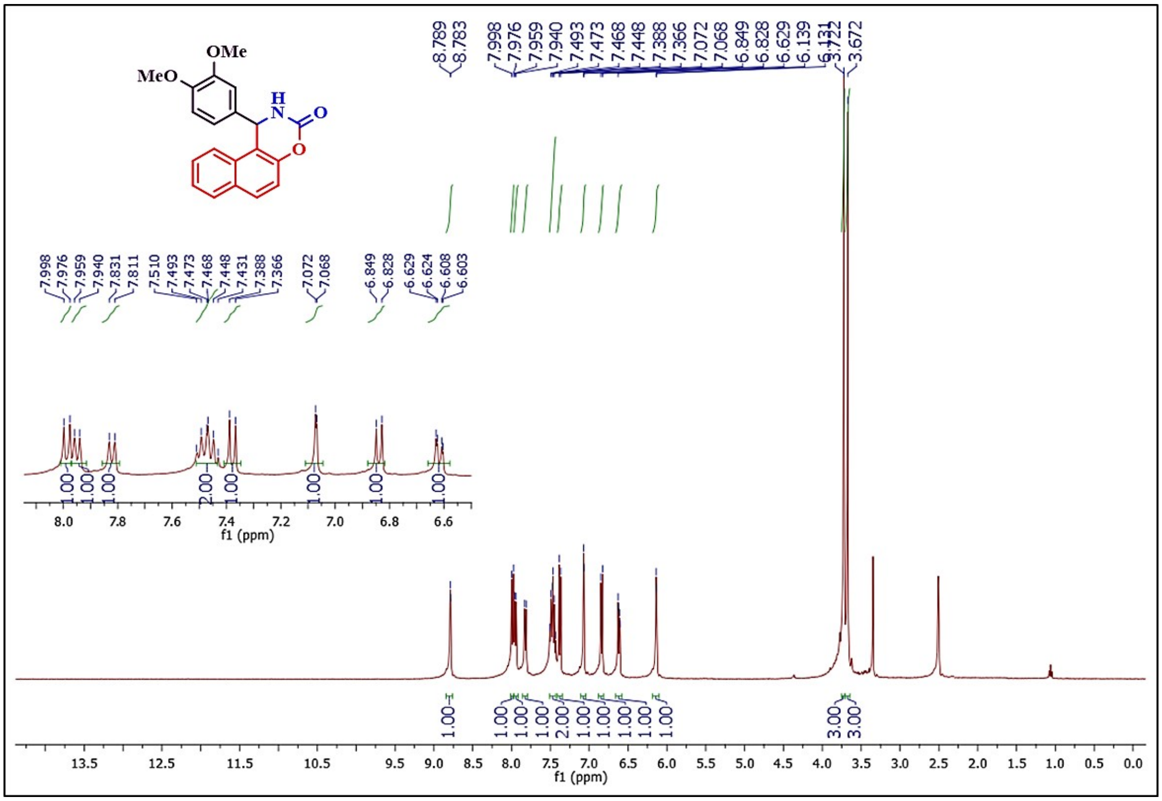

Figure 24: ^{1}H NMR spectrum (400 MHz) of 1-(3,4-Dimethoxyphenyl)-1,2-dihydro-naphtho[1,2-e][1,3]oxazin-3-one **(4k)** in DMSO.

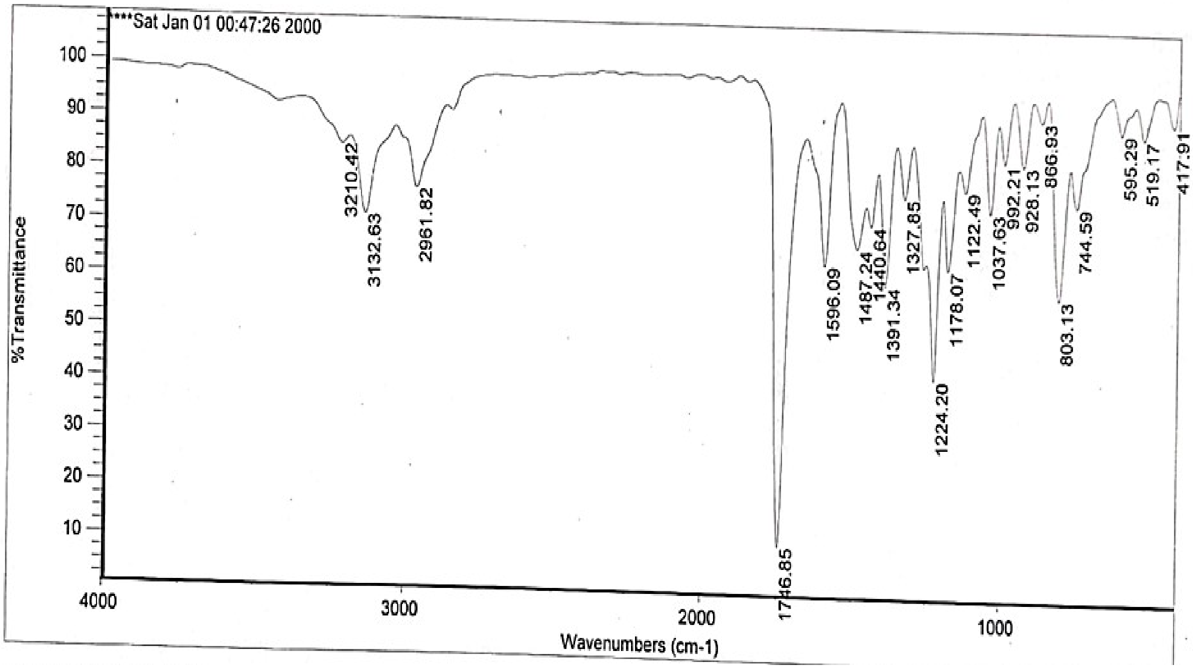

Figure 25: FT-IR spectrum of 1-(3-Methoxyphenyl)-1,2-dihydro-naphtho[1,2-e][1,3] oxazin-3-one **(4l)** in KBr.

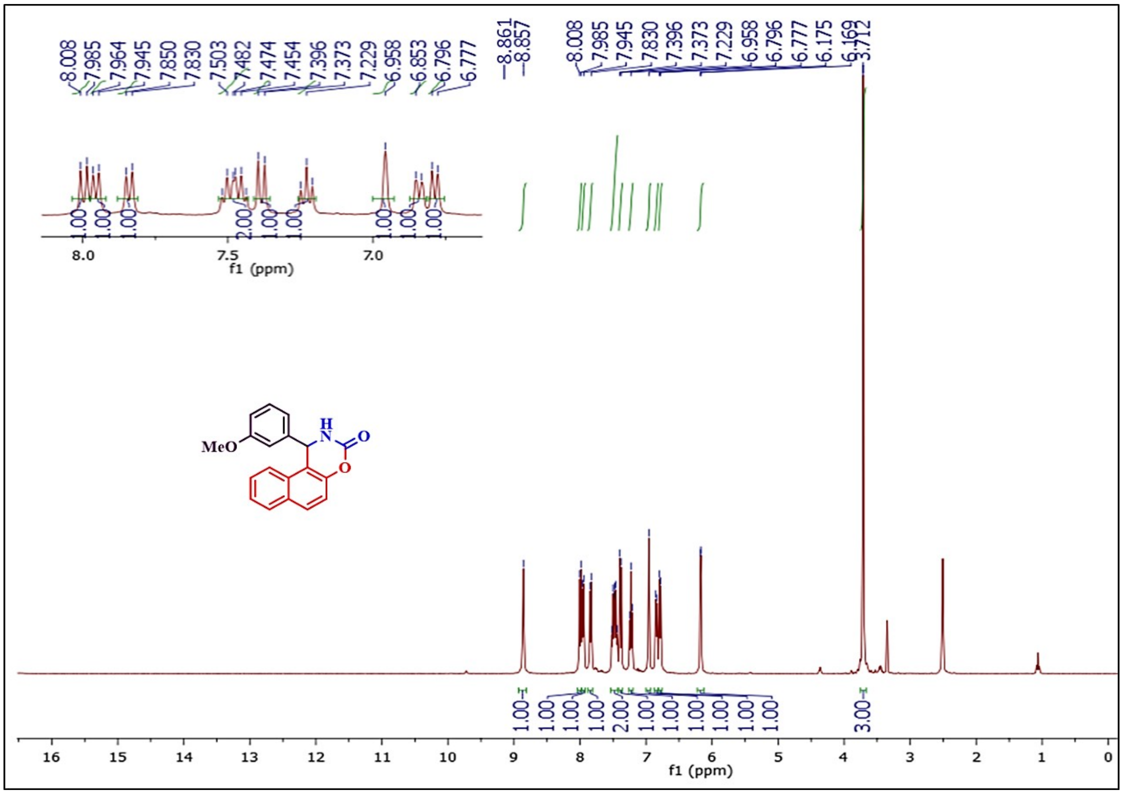

Figure 26: ^{1}H NMR spectrum (400 MHz) of 1-(3-Methoxyphenyl)-1,2-dihydro-naphtho[1,2-e][1,3] oxazin-3-one **(4l)** in DMSO.
